# Early-stage fasting leads to long-term growth inhibition and body composition changes in turbot (*Scophthalmus maximus*)

**DOI:** 10.1016/j.aninu.2025.09.009

**Published:** 2025-11-29

**Authors:** Haiyan Xiong, Dixin Wang, Yuhan Fan, Yanjiao Zhang, Qiang Ma, Yuliang Wei, Mengqing Liang, Houguo Xu

**Affiliations:** a Key Laboratory of Aquaculture Nutrition and Feed (Ministry of Agriculture) & Key Laboratory of Mariculture (Ministry of Education), Ocean University of China, Qingdao 266003, China; b State Key Laboratory of Mariculture Biobreeding and Sustainable Goods, Yellow Sea Fisheries Research Institute, Chinese Academy of Fishery Sciences, Qingdao 266071, China

**Keywords:** Energy mobilization, Feeding regime, Lipid, Nutritional regulation, Starvation

## Abstract

Stimulation of compensatory growth by refeeding after a period of fasting has been used as an economical strategy of growth and body composition manipulation in aquaculture practice. In this study, a total of 300 turbot juveniles, at 85 d post hatch with an initial weight of 5.53 ± 0.06 g, were randomly allocated to 15 tanks (20 fish/tank). The control group was continuously fed, while the four experimental groups were subjected to fasting for 3, 6, 9, or 12 d, respectively, followed by a 60-d refeeding. Each feeding strategy was applied to triplicate tanks. Fish were hand-fed a commercial feed (containing 54.0% protein, 8.0% lipid, and 21.6 MJ/kg gross energy) to apparent satiation. Sampling was conducted at the end of refeeding. Early fasting for 3 to 12 d resulted in final growth retardation compared to the control. This growth inhibition was closely correlated to the fasting duration. The 12-d fasting group showed significantly lower (*P* = 0.027) average weight gain (576%) compared to the control group (709%). Early-stage fasting stimulated the lipid accumulation in various tissues (*P* < 0.05) and the glycogen accumulation in the muscle of fish after refeeding, but reduced the muscle protein and amino acid content (*P* < 0.05). The feeding strategy treatments exerted only minor effects on the fatty acid composition of final fish tissues. The transcriptome analysis confirmed that fasting for 12 d in the juvenile stage still inhibited the peptide and protein biosynthesis even after 60 d of refeeding (*P* < 0.05). In conclusion, compensatory growth through fasting was not observed under the current experimental conditions. Early fasting leads to long-term growth inhibition and changes of body composition in juvenile turbot.

## 1 Introduction

Fasting or feed restriction is naturally occurring in the life cycle of wild fish, as a result of environmental stresses or reproductive processes (Espinoza et al., 2016; Garcia et al., 2018). In modern aquaculture activities, fasting–voluntary or involuntary–also occurs for the purpose of fish handling, health status management, or cost control. In particular, compensatory growth in the refeeding period after fasting has been widely observed, and is demonstrated to have economic benefits in aquaculture.

Compensatory growth is defined as the acceleration of organism growth due to the return of optimal living conditions after a period of feed restriction (Ali et al., 2003; Py et al., 2022). The concept of compensatory growth includes specific cases such as complete or partial compensatory growth, overcompensation, and no compensatory growth depending on the degree of growth compensation. Complete compensatory growth has been observed in previous studies on long-snout catfish (*Leiocassis longirostris*), striped knifejaw (*Oplegnathus fasciatus*), and red seabream (*Pagrus major*) (Mozanzadeh et al., 2021; Oh et al., 2007; Oh and Park, 2019; Zhu et al., 2005), while partial compensatory growth was observed in studies on Siberian sturgeon (*Acipenser baerii*), yellowfin seabream (*Acanthopagrus latus*), Sobaity bream (*Sparidentex hasta*), Korean rockfish (*Sebastes schlegelii*), tongue sole (*Cynoglossus semilaevis*), and rohu (*Labeo rohita*) (Ashouri et al., 2020; Mozanzadeh et al., 2017, 2020; 2021; Oh et al., 2008; Tian et al., 2010; Yengkokpam et al., 2014). As a broader concept, feed restriction includes not only simple fasting, but also reduction of nutritional status, such as reduction of dietary protein content. In these circumstances, partial compensatory growth was observed in Chinese shrimp (*Fenneropenaeus chinensis*), which were refed high-protein (45%) diets for four weeks following being fed with low-protein (15%-30%) diets for two weeks (Wu et al., 2001). It has been demonstrated that moderate fasting or feed restriction could stimulate more efficient utilization of dietary nutrients and consequently spare feed cost. This is economically significant considering that feed is the largest proportion (>60%) of aquaculture cost. In aquaculture practices for redclaw crayfish (*Cherax quadricarinatus*) reared at 27 °C and pacu (*Piaractus mesopotamicus*) reared at 26.3 °C, 50% of the feed can be spared by feed restriction manipulation without compromising the final yield (Favero et al., 2020; Stumpf et al., 2011). Moreover, the reduction of nitrogen and phosphorus emission via feed restriction is also beneficial to the water environment (Bulbul et al., 2014; Debbarma et al., 2020; Hasan and New, 2010).

Despite the benefits of fasting or feeding restriction mentioned above, these benefits often varied depending on species, size, fasting pattern, and environmental factors. In particular, it has been reported that fish in earlier life stages have lower capacity to adapt to feeding restriction (Hvas et al., 2024). However, some previous studies on fish in early-life stages (initial body weight [IBW] from 0.8 to 10.0 g) still demonstrated compensatory growth following feed restriction. Compensatory effects in weight gain, body length, and digestive enzyme activities were observed in yellowfin seabream (*Acanthopagrus latus*, IBW 2.4 g) that underwent fasting for 4 and 8 d followed by refeeding for 16 and 32 d, respectively (Tamadoni et al., 2020). Similar results were observed in another study on yellowfin seabream (IBW 0.8 g), which were restricted to feeding at 6% ration levels for 30 d, followed by normal feeding for another 30 d. Additionally, a study on Sobaity bream (IBW 10.0 g), which were first fed at 0, 25%, 50%, and 75% of the satiation level for a two week period, and then refeeding for six weeks to visual satiation level (Hasanpour et al., 2021; Mozanzadeh et al., 2020). Therefore, when the fasting or feed restriction strategy is applied on juvenile-stage aquaculture animals, the effects should be carefully evaluated (Arendt, 1997; Kirkpatrick and Lofsvold, 1989; Metcalfe, 2003). To date, little information is available regarding the efficacy of different feeding restriction patterns in farmed fish species. Thus, the present study aimed to evaluate the efficacy of different “fasting-refeeding” patterns in an important aquaculture species turbot (*Scophthalmus maximus*), targeting at the juvenile stage, and in terms of regulation of growth, body composition and nutritional metabolism.

## 2 Materials and methods

### 2.1 Animal ethics statement

Fish management and sampling protocols in this experiment were authorized by the Animal Experimental Ethical Inspection of the Animal Care and Use Committee of the Yellow Sea Fisheries Research Institute (No. ACUC202309280529).

### 2.2 Experiment design and fish rearing

**Fig. 1:**
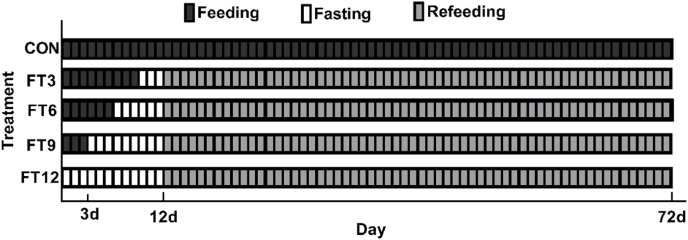
Schematic representation of the five experimental feeding regimes for juvenile turbot (*Scophthalmus maximus*). The control group was continuously fed, while the four experimental groups were subjected to fasting for 3, 6, 9, or 12 d, respectively, followed by a 60-d refeeding (*n* = 3).

Five different feeding regimes were designed. In the control group (CON), fish were fed continuously without fasting. The fish in other groups were fasted for 3 (FT3), 6 (FT6), 9 (FT9), and 12 d (FT12), respectively, which ended on the same day. Following the fasting, all the fish were refed for 60 d (Fig. 1). Each treatment group had three replicate tanks. Each tank was stocked with 20 fish.

A total of 300 turbot juveniles (85 d post hatch, average IBW 5.53 ± 0.06 g) were used in this study. The experimental fish were purchased from Haiyang Huanghai Aquaculture Co., Ltd. (Yantai, Shandong, China) and the feeding experiment was conducted in Yellow Sea Fisheries Research Institute, Chinese Academy of Fishery Sciences (Qingdao, Shandong, China). Fish were reared in polyethylene plastic tanks (57.0 cm × 39.0 cm × 35.0 cm) connected in hydrostatic seawater system. The water was changed by 2/3 each day. A 12 h light:12 h dark photoperiod was established throughout the trial. Before the formal experiment, the experimental fish were fed the same commercial diet for 7 d to acclimate to the experiment conditions. The same commercial diet, which was designed for turbot (Qingdao Surgreen Bioengineering Co., Ltd., Qingdao, Shandong, China; Table 1, Table 2, Table 3), was also used in the following formal feeding trial. The turbots were hand-fed to apparent satiation twice daily (08:00 and 20:00). During the study, the water temperature ranged from 16 to 20 °C, salinity 29 to 30, dissolved oxygen > 7 mg/L, and pH 7.5 to 8.5. The tanks were cleaned at 12:30 of each day, and the residual feeds and feces were siphoned out.

**Table 1:** Proximate composition of the commercial feed used (%, as-is basis).

Nutrients	Concentration
Crude protein	54.0
Crude lipid	8.00
Crude fiber	8.00
Organic matter	73.04
Moisture	12.00
Gross energy, MJ/kg	21.60

**Table 2:** Amino acid compositions of experimental diets (%, dry matter basis).

Items	Concentration
Threonine	2.42
Valine	2.64
Methionine	1.37
Isoleucine	2.42
Leucine	4.19
Phenylalanine	2.13
Lysine	4.34
Histidine	1.69
Argnine	3.08
TEAA	24.28
Aspartic acid	5.10
Serine	2.27
Glutamic acid	8.06
Glycine	3.41
Alanine	3.46
Cysteine	0.21
Tyrosine	1.78
Proline	2.59
TNEAA	26.88

EAA = essential amino acids; NEAA = non-essential amino acids; TEAA = total essential amino acids; TNEAA = total non-essential amino acids.

**Table 3:** Fatty acid composition of experimental diets (%, total fatty acids).

Items	Concentration
C14:0	4.72
C16:0	21.70
C18:0	4.52
SFA	31.00
C16:1n-7	3.90
C18:1n-9	14.95
C20:1n-9	1.84
MUFA	20.69
C18:2n-6	13.03
C20:2n-6	0.26
C20:4n-6	0.73
n-6PUFA	14.03
C18:3n-3	1.85
C20:5n-3	7.11
C22:6n-3	11.12
n-3PUFA	20.07

SFA = saturated fatty acid; MUFA = mono-unsaturated fatty acids; PUFA = poly-unsaturated fatty acids.

### 2.3 Blood and tissue sample collection

At the end of the feeding experiment, fish in all tanks were fasted for 12 h before sampling. Fish were then anesthetized with eugenol, counted, and bulk-weighed. Three fish were randomly selected from each tank, of which the body weight (BW), body length, liver weight, and viscera weight were measured for the calculation of condition factor (*K*), hepatosomatic index (HSI), and viscerosomatic index (VSI). These three fish were then used for the assay of whole-body proximate composition. Six more fish randomly selected from each tank were dissected for the collection of liver, muscle, and subcutaneous adipose tissue near the fin (STF), which were used for the proximate composition analysis. Then, livers from another ten fish per tank were collected for the transcriptome analysis. Blood samples were carefully collected from the caudal vein of six fish. The blood was allowed to clot at ambient temperature for 2 h and at 4 °C for 6 h. After that, centrifugation (1000 × *g* at 4 °C for 10 min) was conducted to separate the supernatants (straw-colored serum samples). All samples collected were immediately frozen with liquid nitrogen, and then stored at −76 °C (the whole fish were stored at −20 °C).

### 2.4 Proximate composition analysis

The proximate composition was analyzed according to AOAC (2005). The moisture content was analyzed by oven-drying samples at 105 °C to constant weight (method 934.01). Crude protein was analyzed by Kjeldahl nitrogen analysis (N × 6.25; method 954.01; KJELTEC 2300, FOSS A/S, Hillerød, Denmark). Ash content was determined by incineration at 550 °C for 8 h (method 942.05). The whole-body crude lipid was analyzed using Soxhlet extraction (method 2003.05; petroleum ether; Soxtec, 2050, FOSS A/S, Hillerød, Denmark), while the lipids in muscle, liver, and STF were extracted using the chloroform-methanol method (Folch et al., 1957). For the experimental diets, the proximate composition (crude protein: method 954.01, Kjeldahl; crude lipid: method 2003.05, Soxhlet; moisture method 934.01; ash: method 942.05; crude fiber: method 978.10) was analyzed by Qingdao Surgreen Bioengineering Co., Ltd. (Qingdao, Shandong, China).

Organic matter (%) = (1 – Moisture – Ash) × 100.

Gross energy was determined using a Parr 6100 oxygen bomb calorimeter (Parr Instrument Co., Moline, IL, USA) following ISO 9831-1998 (ISO, 1998).

### 2.5 Fatty acid compositions of fish tissues

The fatty acid composition of turbot tissues (liver, muscle, and STF) and diets was assayed by gas chromatography (GC2010 pro, Shimadzu Corp., Tokyo, Japan). Briefly, the samples were freeze-dried (FDU-1100, EYELA [Tokyo Rika Kikai Co., Ltd.], Tokyo, Japan) for 48 h, and then the freeze-dried samples were esterified with KOH-methanol and BF_3_-methanol at 75 °C water bath. Fatty acid methyl esters were extracted with hexane, and then subjected to gas chromatography after filtration. The gas chromatography was equipped with a fused silica capillary column (SH-RT-2560, 100 m × 0.25 mm × 0.20 μm) and a flame ionization detector. The column temperature was elevated from 150 to 200 °C by 15 °C/min, and then from 200 to 250 °C by 2 °C/min. The temperature of both injector and detector was 250 °C. The fatty acid contents were expressed as % total fatty acids (% TFA).

### 2.6 Amino acid composition of fish tissues

An automatic amino acid analyzer (Hitachi L-8900 High-Performance Amino Acid Analyzer, Hitachi High-Technologies Corp., Tokyo, Japan) was used to analyze the total amino acid (TAA) composition and free amino acid concentration in the liver, muscle, and diets. For the analysis of TAAs, freeze-dried tissue samples were hydrolyzed in 6 mol/L HCl (15 mL) at 110 °C for 24 h (tryptophan was destroyed under this condition). The solution was then diluted to 50 mL with deionized water, and thereafter the diluted solution (1 mL) was evaporated under a nitrogen stream, dissolved in 0.02 mol/L HCl, filtered, and subjected to the analyzer.

For free amino acids, the lyophilized tissues were deproteinized with trichloroacetic acid of 6%, vortexed for 1 min, and then ultrasonically shaken for 30 min. After that, the samples were centrifuged at 8944 × *g* for 10 min. After evaporation under a nitrogen flow, the sample was dissolved with HCl (0.02 mol/L) and subjected to the automatic amino acid analyzer.

### 2.7 Serum and tissue biochemical indexes

The triglyceride (TG; A110-2-1), total cholesterol (T-CHO; A111-2-1), non-esterified fatty acid (NEFA; A042-2-1), glucose (A154-1-1), pyruvate (A081-1-1), lactate (LD; A019-2-1), glycogen (A043-1-1), total proteins (TP; A045–2), and TAA (A026-1-1) were analyzed using an Infinite M200 microplate reader (Tecan Group Ltd., Männedorf, Switzerland) with commercial kits purchased from Nanjing Jiancheng Bioengineering Institute (Nanjing, Jiangsu, China) according to the manufacturer’s instructions.

### 2.8 Transcriptomics analyses

#### 2.8.1 RNA extraction, library construction, and sequencing

The CON and FT12 groups were selected as the representative groups for the transcriptomic analysis, due to the most significant differences in phenotypic indicators between these two groups. Samples of liver, which is a crucial digestive and metabolic organ in fish, were used for the transcriptomic analysis. Total RNA from liver (liver samples from ten fish per tank were pooled into one sample) was extracted using TRIzol following the manufacturer’s instructions (TRI-Reagent, Ambion, Thermo Fisher Scientific Inc., Austin, TX, USA). RNA integrity and total amount were accurately detected using Agilent 5400 (Agilent Technologies Inc., Santa Clara, CA, USA). All samples had RNA integrity number (RIN) values ≥ 9 and total amount of RNA in all samples > 20 μg. For each group, six separate sequencing libraries were prepared (three biological replicates per treatment group) using the Fast RNA-seq Lib Prep Kit V2 (RK20306, ABclonal Technology Co., Ltd., Wuhan, Hubei, China). The libraries were amplified with 12 cycles of PCR under the following program: initial denaturation at 98 °C for 45 s; then 12 cycles of (98 °C for 10 s, 60 °C for 15 s, and 72 °C for 30 s); followed by a final extension at 72 °C for 1 min. The sequencing of the six libraries that passed the quality test was done using the Illumina NovaSeq X Plus platform (Novogene Co., Ltd., Beijing, China) to obtain reads of 2 × 150 bp.

#### 2.8.2 Identification and enrichment analysis of differentially expressed genes

The measured image data by sequencing were converted into sequence data (reads) by the Consensus Assessment of Sequence and Variation (CASAVA) tool (version 2.19; Illumina Inc., San Diego, CA, USA; https://usermanual.wiki/Document/bcl2fastq2guide15051736v2.164202932.pdf). To ensure the quality and reliability of the data analysis, the raw data were processed by fastp (version 0.19.7) software to remove reads with adapters, low-quality reads, and reads containing ploy-N, after which “clean data” were obtained. Meanwhile, the GC content, Q20 and Q30 of the clean data were calculated.

The six sequenced libraries had 40 to 49 million clean reads with Q20 > 97.0% and Q30 > 94.0%. Clean reads from each of the six libraries after quality control were mapped to turbot reference genome ASM1334776v1 by HISAT2 (version 2.0.5) software, and new transcripts were assembled with the StringTie (Pertea et al., 2015) software. The percentage of sequences aligned to the reference genome of turbot was > 90%. This suggested that the sequencing had a high accuracy. Then, the transcripts of each treatment were quantified per library to obtain fragments per kilobase per million (FPKM) using featureCounts (1.5.0-p3). Differentially expressed genes (DEGs) between the two experimental groups were evaluated using DESeq2 (Anders and Huber, 2010) software and genes were identified as differential when |log_2_ (fold change)| > 0 and *P*-value < 0.05. Kyoto Encyclopedia of Genes and Genomes (KEGG) pathway enrichment analysis and Gene Ontology (GO) functional enrichment analysis of DEGs were performed by ClusterProfiler (version 3.8.1) software. The sequences obtained from the transcriptomic analysis have been submitted to the GenBank of NCBI under the accession number PRJNA1093162.

### 2.9 Evaluation of growth, feed efficiency, and somatic indexes

Weight gain (WG; %) = (Final body weight [FBW] - IBW)/IBW × 100;

Specific growth rate (SGR; %/d) = [ln (FBW, g) - ln (IBW, g)]/Number of days × 100;

Feed intake (g/fish) = Amount of feed consumed (g)/Number of fish.

Survival (%) = Final fish number/Initial fish number × 100;

Feed conversion ratio (FCR) = Feed intake/Weight gain;

VSI (%) = Wet viscera weight/Fish BW × 100;

HSI (%) = Wet liver weight/Fish BW × 100;

*K* = BW/Body length^3^ × 100.

### 2.10 Statistical analysis

One-way analysis of variance (ANOVA) in SPSS 25.0 (International Business Machines Corp., Armonk, NY, USA) for Windows was used for the data statistics (except for transcriptomics). The Tukey’s multiple range test was used to determine the significant differences among experimental groups. There was significant difference at *P* < 0.05. The mathematical model for analysis of variance in one-way ANOVA experiments is as follows:

*X*_*ij*_ = *μ + a*_*i*_ + *e*_*ij*_,

where *X*_*ij*_ is represented the experimental results; *μ* is the population mean; *a*_*i*_ is the *i*-th treatment effect; *e*_*ij*_ is the experimental error; the *e*_*ij*_ is independent of each other and *e*_*ij*_ ∼ (0, σ^2^).

For parameters where ANOVA revealed significant differences (*P* < 0.05), regression analysis was performed to explore the relationship between fasting duration and the evaluation parameters. Regression model selection was guided by the coefficient of determination (*R*^*2*^) and statistical significance (*P*). Models with a higher *R*^*2*^ were preferred for superior fit. Among models with similar *R*^*2*^ values, the one with lower *P* was selected to ensure a more reliable relationship. The final models are presented in the respective tables.

## 3 Results

### 3.1 Growth performance

At the end of the feeding trial, the FBW, WG, and SGR decreased in numerical values as the number of fasting days increased, with a significant decrease observed in FT12 compared to CON (Table 4). Additionally, the FBW exhibited a significant linear decline with prolonged fasting duration (*P* = 0.001). A borderline significant negative correlation was observed in feed intake and fasting duration (*P* = 0.056). There were no significant differences in HSI, VSI, K, FCR, and survival among all groups (*P* > 0.05). Significant cubic regression was observed in the fasting duration and WG (*P* = 0.002).

**Table 4:** Growth performance and somatic parameters.

Items	Groups^1^					SEM	ANOVA	Regression		
	CON	FT3	FT6	FT9	FT12		*P-*value	Model	R^2^	*P-*value
IBW, g	5.46	5.70	5.42	5.59	5.50	0.050	0.757	/	/	/
FBW, g	44.07^a^	41.02^ab^	40.89^ab^	39.50^ab^	37.18^b^	1.122	0.035	Linear	0.545	0.001
WG, %	708.50^a^	619.63^ab^	654.97^ab^	608.20^ab^	576.27^b^	22.576	0.027	Cubic	0.537	0.022
SGR, %/d	2.90^a^	2.74^ab^	2.81^ab^	2.72^ab^	2.65^b^	0.042	0.026	Quadratic	0.479	0.014
Feed intake, g/fish	25.54	25.05	22.98	22.83	20.91	0.836	0.056	/	/	/
FCR	0.66	0.71	0.65	0.67	0.66	0.011	0.190	/	/	/
HSI, %	1.53	1.35	1.36	1.56	1.50	0.040	0.796	/	/	/
VSI, %	6.03	6.18	5.93	5.52	6.13	0.140	0.575	/	/	/
K, g/cm^3^	3.19	3.19	3.20	3.41	3.31	0.044	0.433	/	/	/
Survival, %	100.00	100.00	100.00	100.00	100.00	/	/	/	/	/

IBW = initial body weight; FBW = final body weight; SGR = specific growth rate; WG = weight gain; FCR = feed conversion ratio; HSI = hepatosomatic index; VSI = viscerosomatic index; *K* = condition factor; SEM = standard error of the mean.

Within a row, means without a common superscript letter differ at *P* < 0.05.

“/” For data without significant difference among groups, no regression analysis was performed.

1 The control group was continuously fed, while the four experimental groups were subjected to fasting for 3, 6, 9, or 12 d, respectively, followed by a 60-d refeeding (*n* = 3).

### 3.2 Proximate composition of fish body and tissues

No significant difference (*P* > 0.05) was observed in the crude protein content in whole body and liver among groups (Table 5). However, the muscle crude protein content of the FT12 group was significantly higher (*P* = 0.009) than that in the FT6 and FT9 groups. Opposite results were observed in the moisture content. The muscle moisture content of the FT12 group was significantly lower (*P* = 0.002) than that in the FT6 and FT9 groups. No significant difference (*P* = 0.644) was observed in the whole-body ash content. In general, the lipid content increased with increasing fasting days, significant differences were observed in the lipid content of whole body, liver, and STF (*P <* 0.05).

**Table 5:** Whole-body and tissue proximate composition of experimental turbot (%, wet weight).

Items	Groups^1^					SEM	ANOVA	Regression		
	CON	FT3	FT6	FT9	FT12		*P-*value	Model	*R*^2^	*P-*value
**Whole fish**										
Moisture	76.65	76.57	77.23	76.53	76.99	0.135	0.987	/	/	/
Crude protein	16.15	15.80	15.98	16.21	15.80	0.086	0.636	/	/	/
Crude lipid	7.05^b^	7.56^ab^	6.94^b^	7.51^ab^	8.27^a^	0.235	0.001	Cubic	0.055	0.493
Ash	3.51	3.82	3.62	3.52	3.59	0.056	0.644	/	/	/
**Muscle**										
Moisture	79.01^ab^	79.12^ab^	79.45^a^	79.54^a^	78.78^b^	0.141	0.002	Cubic	0.449	0.001
Crude protein	18.92^ab^	18.87^ab^	18.57^b^	18.61^b^	19.26^a^	0.124	0.009	Cubic	0.380	0.003
Crude lipid	1.03	1.00	0.99	1.18	1.26	0.054	0.074	/	/	/
**Liver**										
Moisture	67.82	67.75	67.33	67.45	66.85	0.173	0.765	/	/	/
Crude protein	11.00	11.56	11.04	10.95	10.44	0.178	0.220	/	/	/
Crude lipid	8.90^b^	10.70^a^	10.91^a^	9.50^ab^	9.67^ab^	0.373	0.005	Cubic	0.358	0.008
**STF**										
Crude lipid	28.75^bc^	24.43^c^	27.57^bc^	35.04^ab^	35.76^a^	2.198	0.001	Cubic	0.525	0.000

STF = subcutaneous adipose tissue near the fin; SEM = standard error of the mean.

Within a row, means without a common superscript letter differ at *P* < 0.05.

“/” For data without significant difference among groups, no regression analysis was performed.

1 The control group was continuously fed, while the four experimental groups were subjected to fasting for 3, 6, 9, or 12 d, respectively, followed by a 60-d refeeding (*n* = 3).

### 3.3 Fatty acid composition in fish tissues

The fasting in early periods resulted in only very slight changes in the muscle and liver fatty acid compositions (Table 6, Table 7). The muscle 16:0 content in the FT12 group were significantly lower (*P* = 0.005) compared with FT3, FT6, and FT9 (Table 7), while the liver 20:2n-6 content in the FT12 group was significantly higher (*P* = 0.018) compared with FT9 (Table 6).

**Table 6:** Fatty acid compositions in liver of experimental turbot (% total fatty acids).

Items	Groups^1^					SEM	ANOVA	Regression		
	CON	FT3	FT6	FT9	FT12		*P-*value	Model	*R*^2^	*P-*value
C14:0	4.19	4.09	4.13	4.05	3.89	0.051	0.214	/	/	/
C16:0	14.98	15.07	14.90	14.83	14.44	0.109	0.661	/	/	/
C18:0	3.06	2.93	2.90	2.87	3.00	0.035	0.826	/	/	/
SFA	22.23	22.10	21.93	21.74	21.32	0.159	0.287	/	/	/
C16:1n-7	4.31	4.31	4.23	4.34	4.18	0.030	0.513	/	/	/
C18:1n-9	19.97	19.04	19.35	19.39	21.06	0.358	0.144	/	/	/
C20:1n-9	1.73	1.83	1.85	1.91	1.78	0.031	0.055	/	/	/
MUFA	26.01	25.19	25.43	25.64	27.02	0.319	0.320	/	/	/
C18:2n-6	19.06	19.61	19.97	20.36	18.58	0.317	0.181	/	/	/
C20:2n-6	1.82^ab^	1.79^ab^	1.68^ab^	1.55^b^	2.06^a^	0.085	0.018	Cubic	0.332	0.013
C20:4n-6	0.89	0.94	0.91	0.88	0.82	0.020	0.462	/ /	/ /	/ /
n-6PUFA	21.76	22.33	22.55	22.77	21.45	0.247	0.333	/	/	/
C18:3n-3	1.60	1.62	1.59	1.50	1.81	0.051	0.146	/	/	/
C20:5n-3	3.57	3.64	3.78	3.79	3.55	0.051	0.312	/	/	/
C22:6n-3	10.02	10.03	9.98	10.11	9.97	0.023	0.992	/	/	/
n-3PUFA	15.20	15.29	15.35	15.40	15.33	0.034	0.979	/	/	/

SFA = saturated fatty acid; MUFA = mono-unsaturated fatty acids; PUFA = poly-unsaturated fatty acids; SEM = standard error of the mean.

Within a row, means without a common superscript letter differ at *P* < 0.05.

“/” For data without significant difference among groups, no regression analysis was performed.

1 The control group was continuously fed, while the four experimental groups were subjected to fasting for 3, 6, 9, or 12 d, respectively, followed by a 60-d refeeding (*n* = 3).

**Table 7:** Fatty acid compositions in muscle of experimental turbot (% total fatty acids).

Items	Groups^1^					SEM	ANOVA	Regression		
	CON	FT3	FT6	FT9	FT12		*P-*value	Model	*R*^2^	*P-*value
C14:0	2.87	2.32	2.49	2.2	2.62	0.117	0.064	/	/	/
C16:0	20.35^ab^	20.80^a^	20.64^a^	20.59^a^	19.49^b^	0.232	0.005	Quadratic	0.386	0.002
C18:0	5.09	5.39	5.42	5.57	4.96	0.113	0.312	/	/	/
SFA	28.30^ab^	28.51^a^	28.55^a^	28.36^a^	27.08^b^	0.274	0.012	Quadratic	0.346	0.006
C16:1n-7	2.57	2.41	2.48	2.21	2.69	0.081	0.539	/	/	/
C18:1n-9	13.55	12.90	13.38	12.97	13.88	0.183	0.242	/	/	/
C20:1n-9	1.19	1.12	1.16	1.09	1.30	0.036	0.494	/	/	/
MUFA	17.31	16.43	17.02	16.27	17.86	0.291	0.356	/	/	/
C18:2n-6	10.51	9.89	10.43	10.22	11.05	0.191	0.126	/	/	/
C20:2n-6	0.59	0.60	0.59	0.55	0.63	0.013	0.094	/	/	/
C20:4n-6	1.12	1.22	1.15	1.25	1.10	0.029	0.109	/	/	/
n-6PUFA	12.22	11.70	12.17	12.02	12.78	0.176	0.115	/	/	/
18:3n-3	1.32	1.28	1.31	1.22	1.33	0.020	0.456	/	/	/
C20:5n-3	7.09	7.30	7.00	7.29	7.28	0.062	0.108	/	/	/
C22:6n-3	19.56	20.85	19.90	20.92	18.94	0.380	0.332	/	/	/
n-3PUFA	27.97	29.43	28.21	29.43	27.55	0.387	0.316	/	/	/

SFA = saturated fatty acid; MUFA = mono-unsaturated fatty acids; PUFA = poly-unsaturated fatty acids; SEM = standard error of the mean.

Within a row, means without a common superscript letter differ at *P* < 0.05.

“/” For data without significant difference among groups, no regression analysis was performed.

1 The control group was continuously fed, while the four experimental groups were subjected to fasting for 3, 6, 9, or 12 d, respectively, followed by a 60-d refeeding (*n* = 3).

In the STF (Table 8), fasting decreased the final 14:0 content, which was significantly lower (*P* = 0.001) in FT9 and FT12 compared to CON. Compared with FT3 and FT6, the 18:2n-6 content in the FT12 group were significantly higher (*P* = 0.029), but the 22:6n-3 (DHA) content was significantly lower (*P* = 0.011).

**Table 8:** Fatty acid compositions in subcutaneous adipose tissue around the fin of experimental turbot (% total fatty acids).

Items	Groups^1^					SEM	ANOVA	Regression		
	CON	FT3	FT6	FT9	FT12		*P-*value	Model	*R*^2^	*P-*value
C14:0	5.11^a^	5.07^ab^	5.01^ab^	4.88^bc^	4.78^bc^	0.061	0.001	Quadratic	0.501	<0.001
C16:0	16.37	16.60	16.59	16.40	16.44	0.048	0.718	/	/	/
C18:0	2.53	2.54	2.64	2.59	2.55	0.020	0.139	/	/	/
SFA	24.01	24.21	24.23	23.87	23.78	0.090	0.184	/	/	/
C16:1n-7	5.30	5.69	5.54	5.63	5.53	0.066	0.353	/	/	/
C18:1n-9	17.13^ab^	16.90^b^	17.08^ab^	17.16^ab^	17.48^a^	0.946	0.029	Cubic	0.309	0.015
C20:1n-9	1.98^ab^	1.91^b^	1.95^ab^	1.98^ab^	2.03^a^	0.020	0.016	Quadratic	0.295	0.002
MUFA	24.42	24.50	24.57	24.76	25.04	0.111	0.073	/	/	/
C18:2n-6	13.59^ab^	12.95^b^	13.24^b^	13.49^ab^	14.34^a^	0.233	0.006	Quadratic	0.388	0.001
C20:2n-6	0.66	0.68	0.66	0.62	0.70	0.013	0.087	/	/	/
C20:4n-6	0.65	0.65	0.64	0.66	0.65	0.003	0.497	/	/	/
n-6PUFA	14.9^ab^	14.29^b^	14.54^b^	14.77^ab^	15.69^a^	0.237	0.006	Quadratic	0.381	0.001
C18:3n-3	1.77	1.75	1.79	1.77	1.74	0.009	0.778	/	/	/
C20:5n-3	7.32^a^	7.28^a^	7.11^ab^	7.22^ab^	6.98^b^	0.062	0.003	Cubic	0.329	0.010
C22:6n-3	11.51^ab^	11.80^a^	11.51^ab^	11.83^a^	11.25^b^	0.107	0.011	Quadratic	0.322	0.003
n-3PUFA	20.61^a^	20.83^a^	20.41^ab^	20.82^a^	19.98^b^	0.158	0.002	Quadratic	0.222	0.026

SFA = saturated fatty acid; MUFA = mono-unsaturated fatty acids; PUFA = poly-unsaturated fatty acids; SEM = standard error of the mean.

Within a row, means without a common superscript letter differ at *P* < 0.05.

“/” For data without significant difference among groups, no regression analysis was performed.

1 The control group was continuously fed, while the four experimental groups were subjected to fasting for 3, 6, 9, or 12 d, respectively, followed by a 60-d refeeding (*n* = 3).

### 3.4 Amino acid compositions in fish tissues

Early fasting significantly reduced (*P <* 0.05) total essential amino acid content expressed (% dry matter) in muscle (Table 9). The fasting significantly reduced (*P* < 0.05) the contents of valine, isoleucine, leucine, and lysine in muscle. The leucine and lysine contents decreased with increasing fasting days. In addition, the content of all detected non-essential amino acids (NEAA) decreased with the prolongation of fasting days, and reached a minimum value in the 12-d fasting group. Compared to the CON group, the NEAA content, except glycine and cysteine, in FT12 significantly decreased (*P* < 0.05). Most amino acids (including both essential and NEAA) exhibited a quadratic response to fasting duration, indicating an initial increase followed by a decrease in their content with increasing fasting duration. A few amino acid parameters, such as phenylalanine and the total non-essential amino acids (TNEAA), showed a linear decline with increasing fasting duration.

**Table 9:** Amino acid composition in the muscle of experimental turbot (%, dry matter).

Items	Groups^1^					SEM	ANOVA	Regression		
	CON	FT3	FT6	FT9	FT12		*P-*value	Model	*R*^2^	*P-*value
Threonine	3.55^ab^	3.62^a^	3.56^ab^	3.48^bc^	3.42^b^	0.035	<0.001	Quadratic	0.596	<0.001
Valine	3.67^a^	3.26^c^	3.35^b^	3.50^b^	3.48^b^	0.070	<0.001	Quadratic	0.434	0.001
Methionine	2.41	2.18	2.22	2.36	2.27	0.043	0.252	/	/	/
Isoleucine	3.39^a^	2.97^c^	3.09^bc^	3.21^b^	3.22^b^	0.070	<0.001	Quadratic	0.452	<0.001
Leucine	6.22^a^	6.03^ab^	5.93^bc^	5.88^bc^	5.80^c^	0.072	<0.001	Quadratic	0.659	<0.001
Phenylalanine	3.24^b^	3.21^b^	3.18^ab^	3.09^a^	3.09^a^	0.031	0.001	Linear	0.477	<0.001
Lysine	7.30^a^	7.06^b^	6.99^bc^	6.93^bc^	6.83^c^	0.079	<0.001	Quadratic	0.659	<0.001
Histidine	1.61^bc^	1.67^a^	1.64^a^	1.58^c^	1.56^c^	0.020	<0.001	Quadratic	0.474	<0.001
Argnine	4.70^a^	4.71^a^	4.59^ab^	4.44^b^	4.41^b^	0.063	<0.001	Quadratic	0.587	<0.001
TEAA	36.10^a^	34.72^b^	34.54^b^	34.48^b^	34.07^b^	0.346	<0.001	Quadratic	0.517	<0.001
Aspartic acid	7.77^ab^	7.95^a^	7.80^ab^	7.62^bc^	7.50^c^	0.077	<0.001	Quadratic	0.555	<0.001
Serine	3.32^bc^	3.42^a^	3.33^ab^	3.23^cd^	3.17^b^	0.043	<0.001	Quadratic	0.622	<0.001
Glutamic acid	12.86^a^	12.87^a^	12.57^ab^	12.31^bc^	12.08^c^	0.154	<0.001	Quadratic	0.709	<0.001
Glycine	3.69	3.65	3.64	3.58	3.57	0.022	0.743	/	/	/
Alanine	4.62^a^	4.59^ab^	4.53^ab^	4.49^ab^	4.44^b^	0.033	0.008	Linear	0.421	<0.001
Cysteine	1.36	1.25	1.27	1.29	1.27	0.019	0.474	/	/	/
Tyrosine	2.85^a^	2.69^b^	2.67^b^	2.67^b^	2.65^b^	0.037	<0.001	Quadratic	0.583	<0.001
Proline	2.70^a^	2.67^ab^	2.57^ab^	2.56^ab^	2.54^b^	0.032	0.007	Quadratic	0.423	0.001
TNEAA	39.19^a^	39.08^a^	38.38^ab^	37.76^b^	37.21^b^	0.380	<0.001	Linear	0.599	<0.001
BCAA	13.29^a^	12.26^b^	12.36^b^	12.60^b^	12.49^b^	0.181	<0.001	Quadratic	0.512	<0.001
TAA	75.29^a^	73.80^ab^	72.92^bc^	72.23^bc^	71.28^c^	0.684	<0.001	Linear	0.578	<0.001

EAA = essential amino acids; NEAA = non-essential amino acids; TEAA = total essential amino acids; TNEAA = total non-essential amino acids; BCAA = branched chain amino acids (isoleucine, leucine, and valine); TAA = total amino acid; SEM = standard error of the mean.

Within a row, means without a common superscript letter differ at *P* < 0.05.

“/” For data without significant difference among groups, no regression analysis was performed.

1 The control group was continuously fed, while the four experimental groups were subjected to fasting for 3, 6, 9, or 12 d, respectively, followed by a 60-d refeeding (*n* = 3).

Moreover, early fasting for 3 to 9 d increased the content of free amino acids in muscle, while the FT12 group showed decreasing trends (Table 10). The FT12 group showed significantly lower (*P <* 0.05) free threonine and glutamic acid contents than the FT3, FT6, and FT9 groups. Most free amino acids displayed a significant quadratic response to fasting duration.

**Table 10:** Free amino acid composition in the muscle of experimental turbot (μg/g, dry matter).

Items	Groups^1^					SEM	ANOVA	Regression		
	CON	FT3	FT6	FT9	FT12		*P-*value	Model	*R*^2^	*P-*value
Taurine acid	11225.36^ab^	11534.82^a^	12191.27^a^	11797.28^a^	10098.65^b^	355.067	0.004	Quadratic	0.374	0.001
Aspartic acid	85.53^b^	85.01^b^	105.45^a^	93.24^ab^	65.29^c^	6.546	0.009	Quadratic	0.353	0.002
Threonine	436.24^ab^	529.39^a^	501.12^a^	533.48^a^	361.73^b^	32.680	0.001	Quadratic	0.392	0.001
Serine	964.97^c^	1273.60^ab^	1147.89^abc^	1352.87^a^	1059.95^bc^	70.106	0.001	Quadratic	0.331	0.003
Glutamic	1144.85^c^	1313.28^ab^	1319.38^ab^	1360.12^a^	1202.87^bc^	40.362	0.011	Quadratic	0.344	0.002
Glycine	3013.31^b^	2981.04^b^	2829.43^b^	3553.35^a^	2865.29^b^	130.809	0.008	Cubic	0.265	0.033
Alanine	1398.89^ab^	1473.27^ab^	1516.92^ab^	1541.83^a^	1319.7^b^	40.699	0.030	Quadratic	0.265	0.011
Cysteine	312.05^ab^	318.14^ab^	416.43^a^	418.96^a^	265.80^b^	30.532	0.002	Quadratic	0.317	0.004
Valine	634.52^ab^	778.24^a^	620.28^ab^	763.87^ab^	520.82^b^	48.116	0.029	Quadratic	0.151	0.093
Methionine	50.02^a^	88.28^b^	143.59^c^	160.00^c^	76.96^b^	20.731	<0.001	Quadratic	0.611	<0.001
Isoleucine	43.56^b^	68.18^ab^	70.99^ab^	96.73^a^	42.75^b^	10.015	0.020	Quadratic	0.215	0.030
Leucine	67.55^b^	101.74^ab^	114.39^a^	110.81^a^	62.89^b^	10.940	0.004	Quadratic	0.413	<0.001
Tyrosine	95.98^b^	134.38^ab^	123.72^ab^	155.64^a^	80.16^b^	13.481	0.006	Quadratic	0.276	0.009
Phenylalanine	104.38	114.17	106.02	145.27	146.72	9.410	0.103	/	/	/
Lysine	243.25^b^	390.53^a^	363.41^ab^	328.84^ab^	316.16^ab^	24.986	0.039	Quadratic	0.216	0.029
Histidine	219.11	230.26	257.18	289.25	194.92	16.228	0.261	/	/	/
Argnine^2^	339.15	445.80	484.78	475.38	357.91	30.265	0.032	Quadratic	0.311	0.005
Proline	1619.78^b^	1755.89^b^	1851.99^ab^	2295.03^a^	1641.00^b^	122.903	0.005	Cubic	0.346	0.007
BCAA	750.71^ab^	963.07^a^	803.31^ab^	974.08^a^	629.94^b^	65.315	0.001	Quadratic	0.290	0.007

BCAA = branched chain amino acids (isoleucine, leucine, and valine); TAA = total amino acid; SEM = standard error of the mean.

Within a row, means without a common superscript letter differ at *P* < 0.05.

“/” For data without significant difference among groups, no regression analysis was performed.

1 The control group was continuously fed, while the four experimental groups were subjected to fasting for 3, 6, 9, or 12 d, respectively, followed by a 60-d refeeding (*n* = 3).

2 For argnine, no significant difference was observed among groups during Tukey’s multiple range test although the *P*-value was < 0.05 in ANOVA.

There were no significant differences in both total (Table S1) and free (Table S2) amino acid compositions in the liver among groups (*P* > 0.05).

### 3.5 Biochemical parameters in the tissues

#### 3.5.1 Lipid metabolism-related biochemical parameters

The serum T-CHO concentration in the FT12 group was significantly higher (*P* = 0.003) compared to other groups, but the liver T-CHO concentration in FT12 was significantly lower (*P* = 0.018) compared to group CON and FT6 (Table 11). Inter-group comparisons (ANOVA) revealed significant differences in the TG content of STF (*P* < 0.001) and muscle (*P* < 0.001), but not in those of serum (*P* = 0.156) or liver (*P* = 0.688). Despite the non-significant regression, the serum TG content showed an apparent increase with prolonged fasting duration.

**Table 11:** Lipid metabolism-related biochemical parameters in serum, liver, muscle, and subcutaneous adipose tissue near the fin (STF) of experimental turbot.

Items	Groups^1^					SEM	ANOVA	Regression		
	CON	FT3	FT6	FT9	FT12		*P-*value	Model	*R*^2^	*P-*value
**Serum, mmol/g**										
TG	18.80	29.39	35.24	36.41	38.68	3.572	0.156	/	/	/
T-CHO	9.63^b^	8.96^b^	8.90^b^	10.23^b^	14.62^a^	1.066	0.003	Cubic	0.267	0.040
NEFA	1.31	0.63	1.09	1.25	1.49	0.146	0.774	/	/	/
TBA, μmol/L	2.03	0.39	0.70	1.71	0.83	0.314	0.127	/	/	/
**Liver, μmol/g**										
TG	88.16	88.97	81.79	80.18	86.29	1.745	0.688	/	/	/
T-CHO	9.33^a^	8.47^ab^	9.38^a^	8.70^ab^	7.52^b^	0.339	0.018	Cubic	0.217	0.018
NEFA	32.68	40.31	38.25	33.16	38.45	1.535	0.068	/	/	/
**Muscle, μmol/g**										
TG	6.19^a^	4.47^c^	5.56^ab^	5.98^b^	4.91^ab^	0.325	<0.001	Cubic	0.425	<0.001
**STF, μmol/g**										
TG	9.98^c^	11.18^bc^	11.12^bc^	12.62^ab^	14.26^a^	0.738	<0.001	Linear	0.472	<0.001
NEFA	0.33	0.25	0.28	0.27	0.25	0.015	0.704	/	/	/

TG = triglyceride; T-CHO = total cholesterol; NEFA = non-esterified fatty acid; TBA = total bile acids; SEM = standard error of the mean.

Within a row, means without a common superscript letter differ at *P* < 0.05.

“/” For data without significant difference among groups, no regression analysis was performed.

1 The control group was continuously fed, while the four experimental groups were subjected to fasting for 3, 6, 9, or 12 d, respectively, followed by a 60-d refeeding (*n* = 3).

Regression analysis further delineated the patterns of change relative to fasting duration: a linear increase in the STF (*P* < 0.001) and a cubic pattern in the muscle (*P* < 0.001).

There was no significant difference in tissue NEFA content among groups (*P* > 0.05).

#### 3.5.2 Glucose metabolism-related biochemical parameters

No significant differences (*P* > 0.05) were observed in serum glucose and liver glycogen contents (Table 12). The muscle glycogen content also showed no significant differences among groups (ANOVA, *P* = 0.353). However, it followed a significant quadratic pattern (*P* = 0.013) in response to fasting. In contrast, the glycogen content in the STF exhibited significant variation among groups (*P* = 0.001), fitting a cubic regression model (*P* = 0.006). The FT12 group showed higher serum pyruvate content than the FT3 group (*P* = 0.052). In liver and muscle, the pyruvate content showed no significant differences among groups (ANOVA, *P* > 0.05). However, a significant quadratic regression (*P* = 0.026) was identified for muscle pyruvate over the fasting period. In general, significant changes in lactate content were detected in the liver (linear decrease, *P* = 0.001) and serum (cubic increase, *P* = 0.001) in response to fasting. In contrast, the muscle lactate content showed no significant variation (*P* = 0.124) among groups, with the FT12 group having the lowest concentration.

**Table 12:** Glucose metabolism-related biochemical parameters in serum, liver, muscle, and subcutaneous adipose tissue near the fin (STF) of experimental turbot.

Items	Groups^1^					SEM	ANOVA	Regression		
	CON	FT3	FT6	FT9	FT12		*P-*value	Model	*R*^2^	*P-*value
**Serum**										
Glucose, mmol/L	3.50	2.77	3.39	3.70	2.76	0.189	0.791	/	/	/
Pyruvate, μmol/mL	0.26	0.20	0.28	0.29	0.30	0.018	0.052	/	/	/
Lactate, mmol/L	8.70^b^	8.37^b^	8.80^b^	10.74^ab^	12.37^a^	0.766	0.002	Cubic	0.474	0.001
**Liver**										
Glycogen, mg/g tissue	7.48	7.05	5.83	6.82	7.56	0.311	0.109	/	/	/
Pyruvate, μmol/g tissue	5.72	6.15	4.18	4.17	5.17	0.400	0.156	/	/	/
Lactate, μmol/g tissue	30.3^a^	27.52^ab^	25.42^b^	25.18^b^	25.64^b^	0.965	0.001	Linear	0.379	0.000
**Muscle**										
Glycogen, mg/g tissue	0.66	0.53	0.73	0.78	0.86	0.057	0.353	/	/	/
Pyruvate, μmol/g tissue	0.21	0.15	0.13	0.12	0.16	0.015	0.121	/	/	/
Lactate, μmol/g tissue	35.45	34.96	37.26	35.17	32.17	0.816	0.124	/	/	/
**STF**										
Glycogen, mg/g tissue	0.28^a^	0.28^a^	0.19^c^	0.21^bc^	0.27^ab^	0.019	0.001	Cubic	0.234	0.006

SEM = standard error of the mean.

Within a row, means without a common superscript letter differ at *P* < 0.05.

“/” For data without significant difference among groups, no regression analysis was performed.

1 The control group was continuously fed, while the four experimental groups were subjected to fasting for 3, 6, 9, or 12 d, respectively, followed by a 60-d refeeding (*n* = 3).

#### 3.5.3 Amino acid metabolism-related biochemical parameters in serum

No difference was observed in TP (*P =* 0.052) and TAA (*P =* 0.357) content in the serum among groups (Table 13).

**Table 13:** Amino acid metabolism-related biochemical parameters in serum of experimental turbot.

Items	Groups^1^					SEM	ANOVA
	CON	FT3	FT6	FT9	FT12		*P-*value
TP, g/L	27.90	29.77	34.02	30.83	31.67	1.015	0.052
TAA, μmol/mL	29.05	24.95	26.53	31.65	29.72	1.186	0.357

TP = total protein; TAA = total amino acid; SEM = standard error of the mean.

1 The control group was continuously fed, while the four experimental groups were subjected to fasting for 3, 6, 9, or 12 d, respectively, followed by a 60-d refeeding (*n* = 3).

### 3.6 Differential gene expression analysis

A total of 735 DEGs were observed (*P* < 0.05) between groups CON and FT12. Compared to CON, FT12 up-regulated the transcription of 368 genes and down-regulated that of 367 genes (Fig. S1 and Tables S3–S5).

**Fig. 2:**
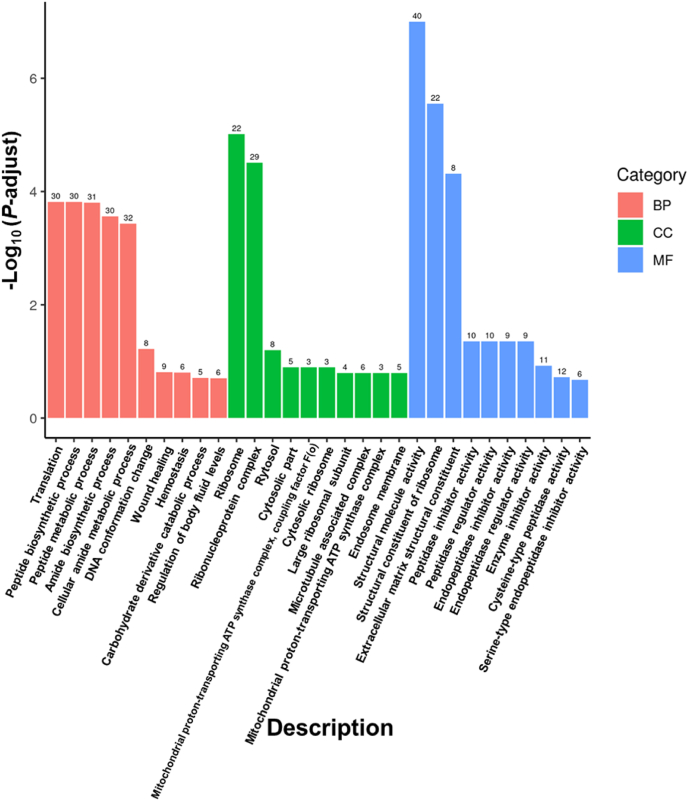
Enriched Gene Ontology (GO) classification of differentially expressed genes (DEGs) between the FT12 and CON groups in juvenile turbot (*Scophthalmus maximus*). The number above the bars represents the number of DEGs enriched in a certain GO term. The CON was control group continuously fed, while the FT12 group was subjected to fasting for 12 d, respectively, followed by a 60-d refeeding (*n* = 3). BP = biological process; CC = cellular component; MF = molecular function.

**Fig. 3:**
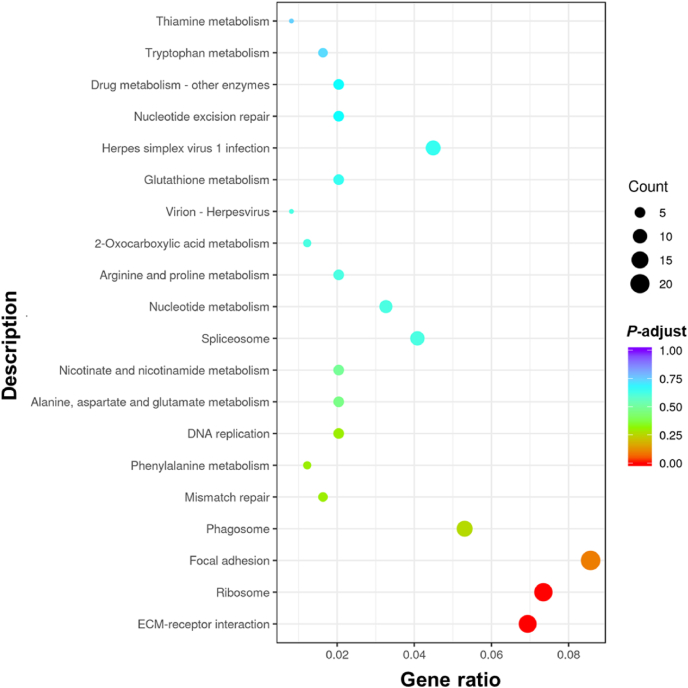
Statistical analysis of Kyoto Encyclopedia of Genes and Genomes (KEGG) pathway enrichment for differentially expressed genes (DEGs) between the FT12 and CON groups in juvenile turbot (*Scophthalmus maximus*). Gene ratio is the ratio of number of DEGs in a certain pathway to number of all annotated genes in this pathway. *P*-adjust is corrected *P*-value by multiple hypothesis test. The spot size represents the number of DEGs enriched in a certain KEGG pathway. The CON was control group continuously fed, while the FT12 group was subjected to fasting for 12 d, respectively, followed by a 60-d refeeding (*n* = 3). ECM = extracellular matrix.

The DEGs were subjected to GO functional enrichment (Fig. 2) and KEGG pathways enrichment analysis (Fig. 3). The DEGs were significantly (*P*-adjust < 0.01) enriched in ten GO terms: peptide biosynthetic process (GO:0043043), peptide metabolic process (GO:0006518), translation (GO:0006412), cellular amide metabolic process (GO:0043603), amide biosynthetic process (GO:0043604), ribosome (GO:0005840), structural molecule activity (GO:0005198), ribonucleoprotein complex (GO:1990904), structural constituent of ribosome (GO:0003735), and extracellular matrix (ECM) structural constituent (GO:0005201). They were significantly (*P* < 0.05) enriched in nine KEGG pathways: ECM-receptor interaction (dre04512), ribosome (dre03010), focal adhesion (dre04510), phagosome (dre04145), mismatch repair (dre03430), phenylalanine metabolism (dre00360), DNA replication (dre03030), alanine, aspartate and glutamate metabolism (dre00250), as well as nicotinate and nicotinamide metabolism (dre00760).

A total of 53 DEGs mapped to these nine KEGG pathways (Table S3), including 40 downregulated genes and 13 upregulated ones in FT12. Nine genes (*col1a1*, *col4a1*, *col1a2*, *col4a2*, *b4gat1*, *vwf*, *lamc3*, *col6a2*, and *col1a1b*) were commonly enriched in ECM-receptor interaction and downregulated to various degrees by FT12 (*P* < 0.05). Genes *sec61g*, *rab5ab*, and *atp6v1e1a*, enriched in the phagosome pathway, were significantly upregulated by FT12 (*P* < 0.05). Among the 14 ribosomal proteins enriched in the ribosome (dre03010) pathway, nine were significantly downregulated by FT12 (*rps29*, *rps28*, *rpl5*, *rpl6*, *rpl8*, *rpl19*, *rpl13*, *rpl24*, and *rps4x*) (*P* < 0.05). Genes *mcm3*, *mcm4*, *mcm6*, *pold1*, *mlh1*, and *exo1*, enriched in the DNA replication and mismatch repair pathways, also showed significant downregulation in FT12 (*P* < 0.05). The genes *got1* and *mao*, enriched in the phenylalanine metabolism pathway, were significantly downregulated by FT12 (*P* < 0.05), but *ddc* in this pathway was significantly upregulated (*P* = 0.047), although to a minor extent. Genes *ppat*, *asrgl1* and *gpt*, enriched in the alanine, aspartate and glutamate metabolism pathway, were significantly downregulated by FT12 (*P* < 0.05). In the nicotinate and nicotinamide metabolism pathway, *pnp6* was significantly downregulated by FT12 (*P* = 0.002). All these results suggested that FT12 profoundly affected the protein synthesis, cell adhesion and migration, signal transduction, phagosome formation and function, as well as the DNA replication in juvenile turbot.

## 4 Discussion

Stimulation of compensatory growth is the main purpose of the application of fasting strategies in aquaculture practices. Fish may have an accelerated growth rate when refed after a period of fasting, thereby facilitating optimized growth and enhancing economic returns. This phenomenon has been observed in previous studies on Nile tilapia (*Oreochromis niloticus*), European sea bass (*Dicentrarchus labrax*), and snow trout (*Schizothorax wangchiachii*) (Abdel-Tawwab et al., 2006; Adakli and Taşbozan, 2015; Wang et al., 2019). These studies endorsed the strategic utilization of fasting to optimize growth and feed utilization in fish farming. However, in the present study, it seemed that this purpose was not achieved. In this study, the fasting in early periods resulted in growth retardation even after 60 d of refeeding, and a general cubic regression was observed in the fasting duration and growth performance.

In fact, discrepancy in compensatory growth when refed after fasting has been widely observed in previous studies on different species under different conditions. Juvenile longsnout catfish (IBW 13.1 g) achieved complete compensatory growth after 28 d of refeeding following 7 d of fasting, while juvenile yellowfin seabream (IBW 4.3 g; refed for 35 d after fasting for 7, 14 or 21 d) and juvenile Korean rockfish (IBW 1.4 g; refed for 35 d after fasting for 10 or 14 d) showed only partial compensatory growth. Partial compensatory growth was even observed in yellowfin seabream with a very low IBW (0.8 g) when refed for 30 d after fasting for 30 d (Mozanzadeh et al., 2020). However, juvenile Nile tilapia (IBW 9.3 g; refed for 7 d after fasting for 5 or 7 d, lasting 60 d periodically) did not show any compensatory growth after refeeding (Mishra et al., 2025).

As indicated above, the occurrence of compensatory growth was probably related to the degree of fasting relative to refeeding. If the fish size was similar among different studies (all fish mentioned above were in the juvenile stage), this degree of fasting relative to refeeding could be associated with both fasting/refeeding days and water temperature, because the water temperature is closely related to the metabolic rate of fish as well as the fish tolerance to fasting. The term “accumulated degree days (ADD)” is a metric used to quantify the cumulative amount of heat energy (temperature over time) available for biological, ecological, or physical processes (Afonin et al., 2024; Crespo et al., 2024; Kelly et al., 2020; Zavell et al., 2024). This term, which is calculated by summing daily temperature deviations from a baseline threshold over a specific period, could be used to partly explain the different results discussed. In feeding studies, the temperature of feeding cessation can be used as the baseline temperature threshold. The low ADD of refeeding duration (114 °C·d) relative to fasting duration (82–144 °C·d) may partly explain the absence of compensatory growth in the study on juvenile Nile tilapia (Mishra et al., 2025) (Table S6). This explanation was also typically suitable for other studies on juvenile Nile tilapia and juvenile tongue sole, which showed that when the ADD of refeeding duration relative to fasting duration decreased, the compensatory growth disappeared (Abdel-Tawwab et al., 2006; Tian et al., 2010). However, the explanation based on ADD may not be suitable for all the studies above. The studies on yellowfin seabream, longsnout catfish, and Korean rockfish did not have higher ADD of refeeding duration relative to fasting duration than the present study, but still showed complete or partial compensatory growth (Mozanzadeh et al., 2020, 2021; Oh et al., 2008; Zhu et al., 2005). The ADDs of the fasting duration in these studies were also higher compared to the present study. Therefore, the higher water temperature itself may also contribute to the stimulation of compensatory growth. Since to date no study has been conducted to specifically investigate the influence of water temperature (as a sole variable) on the compensatory growth during refeeding after fasting, the aforementioned speculations about the roles of water temperature need to validated by further study.

Compensatory growth is also affected by fish age and body size. Fish in early-life stages are sensitive to malnutrition, which could cause various metabolic disorders (Hu et al., 2008; Korosi and Baram, 2010; Love and Williams, 2008; Reul et al., 2015; Weinstock, 2008). Nutritional restriction in newborn animals does not stimulate compensatory growth, but rather leads to permanent growth retardation (China and Holzman, 2014). In the present study, juvenile-stage turbots were used. Although the survival of all fasting groups was 100%, which indicates that three to 12 d of continuous fasting stress was not life-threatening, the fish growth in fasting groups was inhibited after refed. The longer the fasting duration, the worse the growth after refeeding was. These results clearly indicate that larger animals recover more easily after fasting. Relevant previous studies on fish with an IBW > 30.0 g all showed complete or partial compensate growth (Ashouri et al., 2020; Bélanger et al., 2002; Elbialy et al., 2022; Mozanzadeh et al., 2017; Oh et al., 2007; Oh and Park, 2019). Therefore, in aquaculture practices, application of fasting strategies for the purpose of growth stimulation or feed sparing could be more suitable for large-size fish or fish at more developed stages.

Another factor affecting the experimental results could be the lipid content in fish. In general, turbot is a lean fish, with no intraperitoneal fat and a very low lipid level in the carcass (less than 2% of wet weight) (Pasquevich et al., 2011). Considering lipid is the main energy reserve in fish, the insufficient energy reserve in juvenile-stage turbots could make them sensitive to feed deprivation. In contrast, in fat fish such as Atlantic salmon, which have considerable visceral fat, moderate fasting may primarily lead to weight loss of visceral fat, and thus does not substantially reduce the fish growth (Einen et al., 1998). Atlantic salmon even still had normal ranges of muscle lipid and protein level after fasting for 12 weeks (Einen and Thomassen, 1998).

Regarding the change of lipid content via juvenile-stage fasting, in general the lipid content in fish bodies after 60 d of refeeding was increased by a longer prior fasting duration. In another study on turbot, 30 d of fasting (sampling at 0, 10, 20, and 30 d) linearly decreased the lipid content in the liver and STF, but linearly increased the lipid content in the muscle, indicating the mobilization of lipid from storage tissues (liver and STF) to muscle where fatty acid β-oxidation happens (Liu et al., 2021). This suggests that lipid is the primary energy reserve mobilized during feed deprivation. Therefore, the increase of final lipid content in turbot body by juvenile-stage fasting could be a means of fish to deal with potential feed deprivation in the future. In other words, the fasting in early stage had a “memory” effect on fish in following life stages. This “memory” effect was also reflected by the increase of final muscle glycogen content, which serves as another type of energy reserve, as well as by the decrease of lactate content in liver and muscle, which indicates the decreased level of anaerobic glycolysis. These results may provide the fish farmers a method to manipulate the fish body composition, which is closely related to fillet quality. In addition, the increase of final lipid content in turbot body by juvenile-stage fasting was concurrent with the elevated TG levels in the serum and STF, which indicate elevated basic lipid levels in fish body. Similar results were observed in a previous study on rohu, which showed that more lipids were deposited in fish firstly fasted for two to three weeks and then refed for five weeks (Yengkokpam et al., 2014). In Gibel carp (*Carassius auratus gibelio*), the lipid accumulated at a faster rate than dry matter when refed for five weeks after one to two weeks of fasting (Xie et al., 2001). The complete restoration of energy reserves during refeeding after fasting has been observed in several other studies (Heide et al., 2006; Liu et al., 2011, 2021; Morgan and Metcalfe, 2001; Oh et al., 2008).

Nevertheless, in this study, the experimental treatment had only a minor effect on the fatty acid composition of turbot. It has been demonstrated that turbot have different fatty acid preferences for energy mobilization (Xu et al., 2020) in different tissues. The similarity of fatty acid composition among experimental groups in the current study could be due to the fact that the fatty acid composition of farmed fish was mainly determined by the diet, which was the same during the 60 d of refeeding (Xu et al., 2022).

Protein is the last resort for energy mobilization in fish during fasting. Only most of the lipid reserves have been metabolized, begins the energy supply from muscle protein breaking down. In this study, the muscle protein content in the FT6 and FT9 groups decreased, evidencing the low tolerance of juvenile turbot to fasting. Furthermore, the decreased final protein content by juvenile-stage fasting was also concurrent with the decreased amino acid content in the muscle. In particular, some non-essential amino acids, such as isoleucine, leucine and valine, have been reported to be used as energy substrates for maintaining metabolic activities in fish (Duan et al., 2016). Breaking down of muscle protein may reduce the physiological capacities of animals, resulting in serious weakness and even death, which may explain the lack of compensate growth after refeeding in this study.

The transcriptomic analysis has been used as a useful tool to elucidate the physiological changes in response to dietary treatments. In this study, the transcriptomic results confirmed the long-term effects of juvenile-stage fasting on fish. Even after refeeding for 60 d, the 12-d fasting in juvenile stage still resulted in 735 DEGs compared to the control with continuous feeding. It was noteworthy that most of the DEG-enriched GO terms were related to peptide biosynthesis and metabolism.

Similar results were observed in the KEGG enrichment of DEGs. The expression of four fibrillar-forming collagens, *col1a1*, *col4a1*, *col1a2*, and *col4a2,* was downregulated by FT12. Additionally, the expression of integrins *itgb5* and *itgb3a* was also downregulated. Collagen is a major component of the ECM in animal tissues, providing elasticity, mechanical stability, and strength to organs (Gelse, 2003). Focal adhesion, through the binding of integrins to the ECM, forms stable connections between cells and the matrix, regulating normal cell adhesion, migration, and signal transduction. Notably, cell migration plays a crucial role in immune and inflammatory responses (Kanchanawong and Calderwood, 2023; Mishra and Manavathi, 2021). This implies that 12 d of early fasting caused some degree of liver damage and reduced immunity. Genes *sec61g*, *rab5ab*, and *atp6v1e1a*, which positively regulate phagosome maturation, were significantly up-regulated by FT12, indicating the efforts of experimental fish to maintain the cellular homeostasis (Garin et al., 2001).

The minichromosome maintenance (MCM) complex is a replicative helicase necessary for the initiation and extension of “once per cell cycle” DNA replication in eukaryotic cells (Baris et al., 2022; Jenkyn-Bedford et al., 2021; Jones et al., 2021). In this study, FT12 down-regulated the complex-associated genes *mcm3, mcm4, and mcm6*. The highly conserved *actb*, which regulates gene transcription, cell motility, and repair of damaged DNA, was also down-regulated (Schrank et al., 2018). Meanwhile, *pold1*, *mlh1*, and *exo1*, critical genes in the positively regulated DNA mismatch repair pathway (Kadyrov et al., 2006; Lin et al., 2013; Nielsen et al., 2004), were all down-regulated by FT12. These two processes play important roles in maintaining genomic stability. The results above indicate that 12 d of fasting not only affected the genomic stability of turbot but also impaired the immunity and caused a liver damage.

The gene expression of nine ribosomal proteins, *rps29*, *rps28*, *rpl5*, *rpl6*, *rpl8*, *rpl9*, *rpl3*, and *rps4x*, was all down-regulated by FT12. Ribosomal proteins are essential for mRNA translation into protein (Mélèse and Xue, 1995). The present results confirmed the inhibited biosynthesis of peptide and protein in the FT12 group after refeeding. Similar results were observed in a previous fasting study on rainbow trout, which showed that fasting for three weeks down-regulated the gene expression of 47 cytoplasmic ribosomal proteins (30 in the large subunit and 17 in the small subunit) and five mitochondrial ribosomal proteins (two in the large subunit and three in the small subunit) (Salem et al., 2007). Similar results were also observed in previous studies on fine flounder (*Paralichthys adspersus*) (Mendez et al., 2018). Although some ribosomal proteins have been reported as biomarkers sensitive to change of nutritional status such as replacement of fish meal with plant-based ingredients or supplementation of feed additives (De Santis et al., 2015; Kuang et al., 2020; Skugor et al., 2011; Tacchi et al., 2012; Xu et al., 2019), limited fish studies have investigated the nutritional effects on the specific ribosomal proteins mentioned above, which were regulated by the experimental feeding regimes in this study. Further research on fish is needed to elucidate the response of these particular ribosomal proteins to different nutritional conditions. In addition to ribosomal proteins, proteins in other metabolic pathways were also affected by FT12. In the “phenylalanine metabolism” pathway, the expression of *got1* and *mao*, which promote phenylalanine breakdown and the production of monoamine neurotransmitters (Ferrer, 2009; Finberg and Rabey, 2016; Gillman, 2011; Shih et al., 1999; Youdim and Bakhle, 2006), were significantly down-regulated by FT12. The expression of mitochondrial translation release factor in rescue (*mtrfr*), which acts as heterodimer with MTRES1 to prevent aberrant translation by ejecting unfinished nascent chain and peptidyl transporter RNA (tRNA) (Desai et al., 2020), and Y box binding protein 1‌ (*ybx1*), which promotes mRNA stabilization (Yang et al., 2019), were all down-regulated by juvenile-stage fasting, indicating the disturbance of peptide biosynthesis process and metabolic processes (Brandman and Hegde, 2016).

**Fig. 4:**
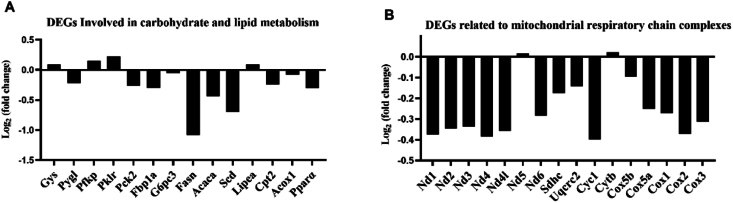
Differentially expressed genes (DEGs) in carbohydrate metabolism, lipid metabolism and mitochondrial respiratory chain complexes in juvenile turbot (*Scophthalmus maximus*) (FT12 vs. CON). (A) DEGs involved in carbohydrate and lipid metabolism. (B) DEGs related to mitochondrial respiratory chain complexes. The CON was control group continuously fed, while the FT12 group was subjected to fasting for 12 d, respectively, followed by a 60-d refeeding (*n* = 3).

Although the gene expression changes related to carbohydrate and lipid metabolism were not so significant, these changes may help to explain the changes of body composition. Therefore, relevant results from the transcriptomics were also examined, including the DEGs involved in carbohydrate and lipid metabolism, as well as those involved in mitochondrial respiratory chain complexes (Fig. 4 and Table S4). The results showed that *pfkp* and *pklr*, which promote glycolysis, were up-regulated, while *pck2*, *fbp1a*, and *g6pc3*, which are involved in gluconeogenesis, were down-regulated. The gene *pygl*, which promotes glycogen breakdown, was also down-regulated, and *gys2*, which promotes glycogen synthesis, was up-regulated by FT12. Genes related to lipid synthesis, such as *fasn*, *acc1*, and *scd*, were down-regulated by FT12 to different degrees, while genes related to lipid breakdown, *abhd6b* and *lipea*, were up-regulated by FT12. Genes involved in fatty acid β-oxidation, *cpt1b*, *acox1*, and *pparα*, were also down-regulated by FT12 to different degrees. These results suggested that in the FT12 group, the glycolysis was active in the fish, while the glycogenolysis was inhibited. Meanwhile, the glycogen synthesis seemed to be promoted, and the gluconeogenesis was suppressed (Fig. 4A). Additionally, FT12 led to lipid synthesis inhibition and lipolysis activation after refed, but unexpectedly led to inhibition of fatty acid β-oxidation. In general, these gene expression results were consistent with the change of body compositions. Furthermore, FT12 significantly up-regulated the nicotinamide nucleotide transhydrogenase 2 (*nnt2*, Gene ID: ENSSMAG00000021288) expression (Table S5), which regulates the balance of NADH and NADPH, subsequently affecting the activity of the tricarboxylic acid cycle. Overexpression of *nnt2* may lead to increased consumption of NADH, resulting in weakened TCA cycle activity, reduced ATP generation, and ultimately insufficient cellular energy supply, consequently affecting normal cellular function (Nesci et al., 2020). Also, *nnt2* is a mitochondrial enzyme, which prompted us to further screen DEGs related to the mitochondrial respiratory chain complexes. The results showed that the DEGs related to the mitochondrial respiratory chain complexes in the FT12 group (*nd1*, *nd2*, *nd3*, *nd4*, *nd4l*, *nd6*, *sdhc*, *uqcrc2*, *cyc1*, *cox5a, cox5b*, *cox1*, *cox2*, and *cox3*) were all down-regulated compared to the control group. These results indicate that fasting for 12 d followed by refeeding ultimately affects the normal function of cells, leading to metabolic disorders and abnormal accumulation of body lipid and muscle glycogen.

All these transcriptomic results clearly indicate that the juvenile-stage fasting has long-term negative effects on the physiological processes of fish in the following life stages, which may well explain the growth retardation after refeeding.

## 5 Conclusions

For juvenile turbot, fasting for three to 12 d resulted in long-term growth retardation after refed for 60 d, and the growth inhibition was closely correlated to the early fasting duration. Compensatory growth was not observed under the current experimental conditions. The juvenile-stage fasting stimulated the lipid accumulation in various tissues and glycogen accumulation in the muscle, but lowered the muscle protein and amino acid contents in final fish body. The fatty acid compositions in the liver, muscle and STF were only marginally affected by different feeding strategies. The transcriptome analysis showed that prolonged fasting (12 d) resulted in metabolic disturbances and inhibited the peptide and protein biosynthesis after 60 d of refeeding, which provides a plausible explanation for the observed growth and body composition outcomes.

## Credit Author Statement

**Haiyan Xiong:** Writing – original draft, Formal analysis, Data curation. **Dixin Wang:** Methodology, Data curation. **Yuhan Fan:** Formal analysis. **Yanjiao Zhang:** Conceptualization. **Qiang Ma:** Software, Methodology. **Yuliang Wei:** Software, Methodology. **Mengqing Liang:** Conceptualization. **Houguo Xu:** Writing – review & editing, Supervision, Funding acquisition, Conceptualization.

## Declaration of competing interest

We declare that we have no financial and personal relationships with other people or organizations that can inappropriately influence our work, and there is no professional or other personal interest of any nature or kind in any product, service and/or company that could be construed as influencing the content of this paper.
