# Supplementary material for: Early-stage fasting leads to long-term growth inhibition and body composition changes in turbot (Scophthalmus maximus)
Source: Anim Nutr. 2025 Nov 29;24:177–91. doi: 10.1016/j.aninu.2025.09.009 (PMC12907870; doi:10.1016/j.aninu.2025.09.009)
Supplement: Multimedia component 1 [file mmc1.docx]

**Table S1** Amino acid composition in the liver of experimental turbot (%, dry matter).

| Items | Groups^1^ | | | | | SEM | *P-*value |
| --- | --- | --- | --- | --- | --- | --- | --- |
|  | CON | FT3 | FT6 | FT9 | FT12 |  |  |
| **EAA** | | | | | | | |
| Threonine | 1.26 | 1.25 | 1.24 | 1.31 | 1.27 | 0.012 | 0.899 |
| Valine | 1.39 | 1.39 | 1.36 | 1.42 | 1.33 | 0.015 | 0.858 |
| Methionine | 0.49 | 0.48 | 0.45 | 0.43 | 0.36 | 0.023 | 0.718 |
| Isoleucine | 1.07 | 1.10 | 1.07 | 1.11 | 1.03 | 0.014 | 0.853 |
| Leucine | 2.02 | 2.02 | 2.03 | 2.11 | 2.03 | 0.017 | 0.930 |
| Phenylalanine | 1.21 | 1.25 | 1.27 | 1.31 | 1.23 | 0.017 | 0.651 |
| Lysine | 2.07 | 2.10 | 2.11 | 2.18 | 2.12 | 0.018 | 0.906 |
| Histidine | 0.67 | 0.69 | 0.69 | 0.72 | 0.68 | 0.008 | 0.749 |
| Argnine | 1.59 | 1.56 | 1.56 | 1.64 | 1.61 | 0.015 | 0.887 |
| TEAA | 11.77 | 11.82 | 11.71 | 12.24 | 11.67 | 0.103 | 0.938 |
| **NEAA** | | | | | | | |
| Aspartic acid | 2.42 | 2.43 | 2.41 | 2.54 | 2.44 | 0.024 | 0.904 |
| Serine | 1.25 | 1.24 | 1.24 | 1.32 | 1.29 | 0.016 | 0.776 |
| Glutamic acid | 3.64 | 3.61 | 3.61 | 3.83 | 3.68 | 0.041 | 0.810 |
| Glycine | 1.28 | 1.23 | 1.23 | 1.31 | 1.35 | 0.023 | 0.612 |
| Alanine | 1.66 | 1.63 | 1.64 | 1.75 | 1.72 | 0.024 | 0.621 |
| Cysteine | 0.78^b^ | 1.00^a^ | 0.99^a^ | 1.02^a^ | 0.97^ab^ | 0.044 | 0.003 |
| Tyrosine | 0.91 | 0.93 | 0.95 | 0.98 | 0.96 | 0.012 | 0.804 |
| Proline | 1.01 | 0.97 | 1.05 | 1.15 | 1.06 | 0.030 | 0.314 |
| TNEAA | 12.96 | 13.04 | 12.70 | 13.70 | 13.48 | 0.182 | 0.758 |
| BCAA | 4.49 | 4.51 | 4.44 | 4.65 | 4.40 | 0.043 | 0.921 |
| TAA | 24.73 | 24.86 | 24.63 | 26.30 | 25.15 | 0.304 | 0.880 |

EAA = essential amino acids; NEAA= non-essential amino acids, TEAA =: total essential amino acids, TNEAA = total non-essential amino acids, BCAA = branched chain amino acids (isoleucine, leucine and valine), TAA = total amino acids.

Within a row, means without a common superscript letter differ at *P* < 0.05.

^1^The control group was continuously fed, while the four experimental groups were subjected to fasting for 3, 6, 9, or 12 d, respectively, followed by a 60-d refeeding (*n* = 3).

^2^Only Cys content showed a significant (*P* ˂ 0.001) quadratic regression with fasting duration, *y* = -0.004*x*^2^ + 0.0616*x* + 0.7912, *R*^2^ = 0.467. No regression analyses were conducted for the remaining parameters owing to non-significant differences among treatments.

**Table S2** Free amino acid composition in the liver of experimental turbot (μg/g, dry matter).

| Items | Groups^1^ | | | | | | SEM | *P-*value |
| --- | --- | --- | --- | --- | --- | --- | --- | --- |
|  | CON | FT3 | | FT6 | FT9 | FT12 |  |  |
| Taurine acid | 6497.01 | | 6364.43 | 6144.26 | 5872.38 | 6084.87 | 109.242 | 0.352 |
| Aspartic acid | 310.60 | | 380.36 | 430.25 | 388.38 | 416.32 | 20.732 | 0.866 |
| Threonine | 630.34 | | 635.12 | 621.10 | 640.16 | 595.12 | 7.959 | 0.992 |
| Serine | 513.56 | | 602.36 | 589.62 | 625.56 | 602.20 | 19.175 | 0.632 |
| Glutamic | 2821.24 | | 2801.64 | 2808.93 | 2852.68 | 2854.75 | 11.020 | 0.999 |
| Glycine | 555.91 | | 586.07 | 563.17 | 590.08 | 589.90 | 7.266 | 0.935 |
| Alanine | 3351.10 | | 3410.34 | 3286.94 | 3428.27 | 3200.78 | 41.808 | 0.883 |
| Cysteine | 423.43 | | 465.86 | 458.44 | 444.42 | 418.60 | 9.321 | 0.958 |
| Valine | 394.66 | | 450.25 | 439.27 | 440.74 | 419.45 | 9.923 | 0.940 |
| Methionine | 249.04 | | 317.03 | 299.62 | 289.61 | 309.81 | 11.933 | 0.816 |
| Isoleucine | 277.40 | | 318.52 | 313.96 | 310.86 | 301.13 | 7.322 | 0.959 |
| Leucine | 627.68 | | 732.10 | 720.60 | 686.10 | 697.78 | 18.204 | 0.962 |
| Tyrosine | 256.20 | | 303.06 | 299.71 | 293.90 | 273.49 | 8.897 | 0.823 |
| Phenylalanine | 415.00 | | 448.55 | 435.30 | 426.89 | 441.22 | 5.810 | 0.992 |
| Lysine | 474.08 | | 545.18 | 552.81 | 511.05 | 528.65 | 14.047 | 0.941 |
| Histidine | 340.87 | | 356.21 | 330.09 | 389.66 | 355.74 | 10.056 | 0.603 |
| Argnine | 488.99 | | 566.39 | 615.55 | 526.79 | 401.83 | 36.231 | 0.406 |
| Proline | 394.94 | | 393.22 | 414.68 | 408.35 | 438.56 | 8.211 | 0.927 |

^1^The control group was continuously fed, while the four experimental groups were subjected to fasting for 3, 6, 9, or 12 d, respectively, followed by a 60-d refeeding (*n* = 3).

**Table S3** Differentially expressed genes (DEGs) in significantly enriched KEGG pathways (*P* < 0.05).

| Genes | Full names | Log_2_ (fold change) | *P*-value | KEGG Pathway |
| --- | --- | --- | --- | --- |
| *Npnt* | Nephronectin | -1.287 | 0.033 | ECM-receptor interaction |
| *Fras1* | Fraser extracellular matrix complex subunit 1 | -1.111 | 0.033 | ECM-receptor interaction |
| *Col1a1* | Collagen type I alpha 1 chain | -0.899 | <0.001 | ECM-receptor interaction; focal adhesion |
| *Col4a1* | Collagen type IV alpha 1 chain | -0.672 | <0.001 | ECM-receptor interaction; focal adhesion |
| *Col1a2* | Collagen type I alpha 2 chain | -0.773 | <0.001 | ECM-receptor interaction; focal adhesion |
| *Col4a2* | Collagen type IV alpha 2 chain | -0.704 | <0.001 | ECM-receptor interaction; focal adhesion |
| *B4gat1* | Beta-1,4-glucuronyltransferase 1 | -0.703 | 0.012 | ECM-receptor interaction; focal adhesion |
| *Vwf* | Von willebrand factor | -0.637 | 0.019 | ECM-receptor interaction; focal adhesion |
| *Lamc3* | Laminin subunit gamma 3 | -1.235 | 0.034 | ECM-receptor interaction; focal adhesion |
| *Col6a2* | Collagen type IV alpha 2 chain | -0.440 | 0.036 | ECM-receptor interaction; focal adhesion |
| *Col1a1b* | Collagen, type I, alpha 1b | -0.851 | <0.001 | ECM-receptor interaction; focal adhesion |
| *Itgb5* | Integrin subunit beta 5 | -0.436 | 0.022 | ECM-receptor interaction; focal adhesion; Phagosome |
| *Itgb3a* | Integrin subunit beta 3 | -0.668 | 0.040 | ECM-receptor interaction; focal adhesion; Phagosome |
| *Flnb* | Filamin B | -0.442 | 0.010 | Focal adhesion |
| *Crk* | V-crk avian sarcoma virus CT10 oncogene homolog | -0.607 | 0.020 | Focal adhesion |
| *Capn2b* | Calpain 2, (m/II) large subunit b | -0.526 | 0.024 | Focal adhesion |
| *Grb2b* | Growth factor receptor-bound protein 2b | -0.583 | 0.034 | Focal adhesion |
| *Rasgrf1* | Ras protein specific guanine nucleotide releasing factor 1 | 2.384 | 0.036 | Focal adhesion |
| *Actb* | Actin beta | -0.362 | 0.046 | Phagosome; focal adhesion |
| *Sec61g* | SEC61 translocon subunit gamma | 0.729 | <0.001 | Phagosome |
| *Rab5ab* | Member rat sarcoma oncogene family, b | 0.528 | 0.020 | Phagosome |
| *Canx* | Calnexin | -0.520 | 0.031 | Phagosome |
| *Atp6v1e1a* | ATPase H^+^ transporting V1 subunit E1a | 0.639 | 0.032 | Phagosome |
| *Rps15a* | Ribosomal protein s15a | 0.577 | <0.001 | Ribosome |
| *Mrps27* | Mitochondrial ribosomal protein s27 | 0.620 | 0.002 | Ribosome |
| *Rps7* | Ribosomal protein s7 | 0.415 | 0.022 | Ribosome |
| *Rplp2* | Ribosomal protein lateral stalk subunit p2 | 0.588 | 0.040 | Ribosome |
| *Rps21* | Ribosomal protein s21 | 0.387 | 0.050 | Ribosome |
| *Rps29* | Ribosomal protein s29 | -1.102 | <0.001 | Ribosome |
| *Rps28* | Ribosomal protein s28 | -0.558 | 0.004 | Ribosome |
| *Rpl5* | Ribosomal protein l5 | -0.518 | 0.008 | Ribosome |
| *Rpl24* | Ribosomal protein l24 | -0.478 | 0.009 | Ribosome |
| *Rpl6* | Ribosomal protein l6 | -0.408 | 0.020 | Ribosome |
| *Rpl8* | Ribosomal protein l8 | -0.374 | 0.028 | Ribosome |
| *Rpl19* | Ribosomal protein l19 | -0.389 | 0.031 | Ribosome |
| *Rpl13* | Ribosomal protein l13 | -0.346 | 0.033 | Ribosome |
| *Rps4x* | Ribosomal protein s4 x-linked | -0.401 | 0.036 | Ribosome |
| *Mcm3* | Minichromosome maintenance complex component 3 | -1.242 | 0.039 | DNA replication |
| *Mcm4* | Minichromosome maintenance complex component 4 | -0.779 | 0.040 | DNA replication |
| *Mcm6* | Minichromosome maintenance complex component 6 | -0.936 | 0.002 | DNA replication |
| *Pold1* | Dna polymerase delta 1 | -0.948 | 0.017 | DNA replication; mismatch repair |
| *Mlh1* | Mutl homolog 1 | -0.594 | 0.035 | Focal adhesion; phagosome; mismatch repair |
| *Exo1* | Exonuclease 1 | -1.242 | 0.039 | Mismatch repair |
| *Got1* | Glutamic-oxaloacetic transaminase 1 | -0.394 | 0.015 | Phenylalanine metabolism; alanine, aspartate and glutamate metabolism |
| *Mao* | Monoamine oxidase | -0.506 | 0.020 | Phenylalanine metabolism |
| *Ddc* | Dopa decarboxylase | 0.379 | 0.047 | Phenylalanine metabolism |
| *Ppat* | Phosphoribosyl pyrophosphate amidotransferase | -0.509 | 0.025 | Alanine, aspartate and glutamate metabolism |
| *Asrgl1* | Asparaginase and isoaspartyl peptidase 1 | -0.587 | 0.046 | Alanine, aspartate and glutamate metabolism |
| *Gpt* | Glutamic-pyruvic transaminase | -0.339 | 0.049 | Alanine, aspartate and glutamate metabolism |
| *Mibp* | Muscle-specific beta 1 integrin binding protein | 1.963 | 0.002 | Nicotinate and nicotinamide metabolism |
| *Pnp6* | Purine nucleoside phosphorylase 6 | -2.042 | 0.002 | Nicotinate and nicotinamide metabolism |
| *Nt5c2l1* | 5-Nucleotidase, cytosolic II, like 1 | 1.150 | 0.015 | Nicotinate and nicotinamide metabolism |
| *Sirt2* | Sirtuin 2 | 0.500 | 0.017 | Nicotinate and nicotinamide metabolism |

FC = fold change; KEGG = Kyoto Encyclopedia of Genes and Genomes; ECM = extracellular matrix; RAS = rat sarcoma oncogene.

**Table S4** Differentially expressed genes (DEGs) in key metabolic pathways: carbohydrate metabolism, lipid metabolism, and mitochondrial respiratory chain.

| Categories | Gene lD | Gene  abbreviations | Full names | Log_2_ (fold change) |
| --- | --- | --- | --- | --- |
| Glycogenesis | ENSSMAG00000014961 | *Gys* | Glycogen synthase | 0.089 |
| Glycogenolysis | ENSSMAG00000012269 | *Pygl* | Phosphorylase, glycogen, liver | -0.222 |
|  | ENSSMAG00000006480 | *Pfkp* | Phosphofructokinase, platelet b | 0.153 |
|  | ENSSMAG00000014175 | *Pklr* | Pyruvate kinase L/R | -0.265 |
| Gluconeogenesis | ENSSMAG00000020067 | *Pck2* | Phosphoenolpyruvate carboxykinase 2 | -0.301 |
|  | ENSSMAG00000013448 | *Fbp1**a* | Fructose-1,6-bisphosphatase 1 a | -0.055 |
|  | ENSSMAG00000008080 | *G6pc3* | Glucose-6-phosphatase catalytic subunit 3 | 0.046 |
| Lipogenesis | ENSSMAG00000018711 | *Fsn* | Fatty acid synthase | -1.100 |
|  | ENSSMAG00000018733 | *Acaca* | Acetyl-CoA carboxylase alpha | -0.441 |
|  | ENSSMAG00000000822 | *Scd* | Stearoyl-CoA desaturase | -0.703 |
| Lipolysis | ENSSMAG00000001823 | *Lipea* | Lipase, hormone-sensitive a | 0.096 |
|  | ENSSMAG00000010436 | *Mgll* | Monoacylglycerol Lipase | 3.060 |
| Fatty acid β-oxidation | ENSSMAG00000005157 | *Cpt2* | Carnitine palmitoyitransferase 2 | -0.243 |
|  | ENSSMAG00000017686 | *Acox1* | Acyl-CoA oxidase 1 | -0.077 |
|  | ENSSMAG00000018220 | *Ppara* | Peroxisome proliferator-activated receptor alpha a | -0.303 |
| Complex I: NADH:Ubiquinone oxidoreductase (CI) | ENSSMAG00000037541 | *Nd1* | NADH dehydrogenase subunit 1 | -0.376 |
|  | ENSSMAG00000029693 | *Nd2* | NADH dehydrogenase subunit 2 | -0.346 |
|  | ENSSMAG00000022131 | *Nd3* | NADH dehydrogenase subunit 3 | -0.336 |
|  | ENSSMAG00000025377 | *Nd4* | NADH dehydrogenase subunit 4 | -0.387 |
|  | ENSSMAG00000037383 | *Nd4l* | NADH dehydrogenase subunit 4L | -0.360 |
|  | ENSSMAG00000021620 | *Nd5* | NADH dehydrogenase subunit 5 | 0.016 |
|  | ENSSMAG00000023056 | *Nd6* | NADH dehydrogenase subunit 6 | -0.284 |
| Complex II: Succinate-ubiquinone Oxidoreductase (CII) | ENSSMAG00000023076 | *Sdhc* | Succinate dehydrogenase complex, subunit c, integral membrane protein | -0.175 |
| Complex III: Ubiquinone-Cytochrome C oxidoreductase (CIII) | ENSSMAG00000010431 | *Uqcrc2* | Ubiquinol-cytochrome c reductase core protein 2 | -0.143 |
|  | ENSSMAG00000002094 | *Cyc1* | Cytochrome c-1 | -0.400 |
|  | ENSSMAG00000037503 | *Cytb* | Cytochrome b | 0.021 |
| Complex IV: Cytochrome C oxidase (CIV) | ENSSMAG00000012569 | *Cox5b* | Cytochrome C oxidase subunit 5B | -0.371 |
|  | ENSSMAG00000010719 | *Cox5a* | Cytochrome C oxidase subunit 5A | -0.313 |
|  | ENSSMAG00000031451 | *Cox1* | Cytochrome C oxidase subunit 1 | -0.096 |
|  | ENSSMAG00000031621 | *Cox2* | Cytochrome C oxidase subunit 2 | -0.252 |
|  | ENSSMAG00000033259 | *Cox3* | Cytochrome C oxidase subunit 3 | -0.273 |

**Table S5** Detailed information about the differentially expressed genes (DEGs).

| Gene IDs | Log_2_ (fold change) | *P*-value | *P*-adjust |
| --- | --- | --- | --- |
| ENSSMAG00000028844 | 8.453 | 0.005 | <0.001 |
| ENSSMAG00000017973 | 4.779 | 0.008 | 0.734 |
| ENSSMAG00000005357 | 4.698 | 0.012 | 0.855 |
| ENSSMAG00000037295 | 4.687 | 0.024 | 0.980 |
| ENSSMAG00000007755 | 4.615 | <0.001 | 1.000 |
| ENSSMAG00000033288 | 4.611 | 0.017 | <0.001 |
| ENSSMAG00000008624 | 4.398 | 0.007 | 1.000 |
| ENSSMAG00000002562 | 4.327 | 0.027 | 0.843 |
| ENSSMAG00000032669 | 4.291 | 0.024 | 1.000 |
| ENSSMAG00000001530 | 4.277 | 0.029 | 1.000 |
| ENSSMAG00000017081 | 4.165 | 0.035 | 1.000 |
| ENSSMAG00000028647 | 4.155 | 0.040 | 1.000 |
| ENSSMAG00000027622 | 4.029 | 0.043 | 1.000 |
| ENSSMAG00000002131 | 4.017 | <0.001 | 1.000 |
| ENSSMAG00000018160 | 3.676 | <0.001 | 0.099 |
| ENSSMAG00000037609 | 3.630 | 0.044 | <0.001 |
| ENSSMAG00000006274 | 3.625 | <0.001 | 1.000 |
| ENSSMAG00000021288 | 3.510 | 0.004 | <0.001 |
| ENSSMAG00000024204 | 3.495 | 0.045 | 0.659 |
| ENSSMAG00000030079 | 3.445 | 0.050 | 1.000 |
| ENSSMAG00000013529 | 3.443 | 0.006 | 1.000 |
| ENSSMAG00000011956 | 3.414 | 0.005 | 0.760 |
| ENSSMAG00000015085 | 3.338 | 0.018 | 1.000 |
| ENSSMAG00000037605 | 3.314 | <0.001 | <0.001 |
| ENSSMAG00000025598 | 3.029 | <0.001 | <0.001 |
| ENSSMAG00000033485 | 2.993 | 0.041 | 1.000 |
| ENSSMAG00000034193 | 2.957 | <0.001 | <0.001 |
| ENSSMAG00000018453 | 2.953 | 0.046 | 1.000 |
| ENSSMAG00000026918 | 2.883 | 0.022 | 1.000 |
| ENSSMAG00000013712 | 2.871 | 0.022 | 1.000 |
| ENSSMAG00000005368 | 2.813 | 0.005 | 0.734 |
| ENSSMAG00000028915 | 2.743 | 0.018 | 1.000 |
| ENSSMAG00000026972 | 2.713 | 0.040 | 1.000 |
| ENSSMAG00000021754 | 2.648 | <0.001 | <0.001 |
| ENSSMAG00000034419 | 2.643 | 0.022 | 1.000 |
| ENSSMAG00000034839 | 2.604 | <0.001 | <0.001 |
| ENSSMAG00000012572 | 2.504 | <0.001 | <0.001 |
| ENSSMAG00000013574 | 2.473 | 0.041 | 1.000 |
| ENSSMAG00000001289 | 2.384 | 0.036 | 1.000 |
| ENSSMAG00000027713 | 2.326 | 0.033 | 1.000 |
| ENSSMAG00000028838 | 2.316 | 0.044 | 1.000 |
| ENSSMAG00000037885 | 2.246 | 0.003 | 0.580 |
| ENSSMAG00000027598 | 2.178 | 0.004 | 0.623 |
| ENSSMAG00000035952 | 2.127 | 0.001 | 0.415 |
| Novel.288 | 2.117 | 0.001 | 0.311 |
| ENSSMAG00000007890 | 2.085 | 0.039 | 1.000 |
| ENSSMAG00000015476 | 2.079 | 0.045 | 1.000 |
| ENSSMAG00000027494 | 2.071 | <0.001 | <0.001 |
| ENSSMAG00000038108 | 2.034 | 0.045 | 1.000 |
| ENSSMAG00000025675 | 2.004 | <0.001 | <0.001 |
| ENSSMAG00000015894 | 1.973 | 0.038 | 1.000 |
| ENSSMAG00000005298 | 1.963 | 0.002 | 0.463 |
| ENSSMAG00000032079 | 1.959 | <0.001 | <0.001 |
| ENSSMAG00000002877 | 1.957 | 0.008 | 0.855 |
| Novel.223 | 1.945 | 0.001 | 0.311 |
| ENSSMAG00000026041 | 1.895 | 0.009 | 0.904 |
| ENSSMAG00000027465 | 1.874 | 0.031 | 1.000 |
| ENSSMAG00000013585 | 1.811 | 0.035 | 1.000 |
| ENSSMAG00000032037 | 1.773 | 0.001 | 0.415 |
| ENSSMAG00000029625 | 1.767 | <0.001 | <0.001 |
| ENSSMAG00000032802 | 1.761 | 0.003 | 0.611 |
| ENSSMAG00000019221 | 1.738 | 0.008 | 0.855 |
| ENSSMAG00000003940 | 1.719 | 0.022 | 1.000 |
| ENSSMAG00000037621 | 1.700 | 0.047 | 1.000 |
| ENSSMAG00000009423 | 1.668 | 0.005 | 0.713 |
| ENSSMAG00000037629 | 1.654 | <0.001 | 0.002 |
| ENSSMAG00000037154 | 1.640 | 0.034 | 1.000 |
| ENSSMAG00000035341 | 1.638 | 0.040 | 1.000 |
| ENSSMAG00000018026 | 1.616 | 0.036 | 1.000 |
| ENSSMAG00000000681 | 1.599 | 0.032 | 1.000 |
| ENSSMAG00000004925 | 1.518 | 0.033 | 1.000 |
| ENSSMAG00000005914 | 1.484 | 0.026 | 1.000 |
| ENSSMAG00000005142 | 1.467 | 0.039 | 1.000 |
| ENSSMAG00000018872 | 1.449 | 0.038 | 1.000 |
| ENSSMAG00000015843 | 1.436 | 0.002 | 0.457 |
| ENSSMAG00000011925 | 1.435 | 0.006 | 0.751 |
| ENSSMAG00000015800 | 1.406 | 0.009 | 0.889 |
| ENSSMAG00000030415 | 1.353 | 0.012 | 0.980 |
| ENSSMAG00000018251 | 1.330 | 0.011 | 0.952 |
| ENSSMAG00000003467 | 1.327 | 0.019 | 1.000 |
| ENSSMAG00000019249 | 1.323 | 0.045 | 1.000 |
| ENSSMAG00000028984 | 1.323 | 0.037 | 1.000 |
| ENSSMAG00000000772 | 1.312 | 0.006 | 0.793 |
| ENSSMAG00000012082 | 1.261 | 0.001 | 0.357 |
| ENSSMAG00000011058 | 1.245 | 0.015 | 1.000 |
| ENSSMAG00000006729 | 1.227 | 0.006 | 0.759 |
| ENSSMAG00000020552 | 1.211 | 0.010 | 0.909 |
| ENSSMAG00000025819 | 1.192 | <0.001 | <0.001 |
| ENSSMAG00000000608 | 1.187 | 0.030 | 1.000 |
| ENSSMAG00000000328 | 1.185 | 0.038 | 1.000 |
| ENSSMAG00000006649 | 1.181 | 0.037 | 1.000 |
| Novel.330 | 1.173 | 0.027 | 1.000 |
| ENSSMAG00000018906 | 1.168 | <0.001 | 0.147 |
| ENSSMAG00000015947 | 1.166 | 0.004 | 0.659 |
| ENSSMAG00000020335 | 1.150 | 0.015 | 1.000 |
| ENSSMAG00000015241 | 1.128 | 0.032 | 1.000 |
| ENSSMAG00000003214 | 1.120 | 0.038 | 1.000 |
| ENSSMAG00000015624 | 1.119 | 0.020 | 1.000 |
| ENSSMAG00000025640 | 1.116 | 0.006 | 0.770 |
| ENSSMAG00000026503 | 1.110 | 0.006 | 0.787 |
| ENSSMAG00000006930 | 1.079 | 0.043 | 1.000 |
| ENSSMAG00000004825 | 1.069 | <0.001 | 0.080 |
| ENSSMAG00000018485 | 1.066 | 0.014 | 1.000 |
| ENSSMAG00000017101 | 1.044 | 0.044 | 1.000 |
| ENSSMAG00000002603 | 1.044 | 0.037 | 1.000 |
| ENSSMAG00000030927 | 1.043 | 0.022 | 1.000 |
| ENSSMAG00000009419 | 1.042 | 0.033 | 1.000 |
| ENSSMAG00000001636 | 1.036 | 0.044 | 1.000 |
| ENSSMAG00000015349 | 1.030 | 0.016 | 1.000 |
| ENSSMAG00000016425 | 1.026 | 0.013 | 1.000 |
| ENSSMAG00000011466 | 1.019 | 0.012 | 0.980 |
| ENSSMAG00000028867 | 0.985 | 0.034 | 1.000 |
| ENSSMAG00000004980 | 0.985 | 0.021 | 1.000 |
| ENSSMAG00000036463 | 0.970 | 0.034 | 1.000 |
| ENSSMAG00000035853 | 0.965 | 0.006 | 0.755 |
| ENSSMAG00000019622 | 0.952 | 0.041 | 1.000 |
| ENSSMAG00000018949 | 0.951 | 0.032 | 1.000 |
| ENSSMAG00000005486 | 0.947 | 0.010 | 0.931 |
| ENSSMAG00000008006 | 0.928 | 0.016 | 1.000 |
| ENSSMAG00000029975 | 0.927 | 0.030 | 1.000 |
| ENSSMAG00000017810 | 0.913 | 0.012 | 0.980 |
| ENSSMAG00000000343 | 0.910 | 0.018 | 1.000 |
| ENSSMAG00000012558 | 0.906 | <0.001 | 0.061 |
| ENSSMAG00000001554 | 0.899 | 0.004 | 0.647 |
| Novel.157 | 0.899 | 0.001 | 0.382 |
| ENSSMAG00000001523 | 0.896 | 0.038 | 1.000 |
| ENSSMAG00000018357 | 0.895 | 0.020 | 1.000 |
| ENSSMAG00000018568 | 0.892 | 0.006 | 0.770 |
| ENSSMAG00000032134 | 0.890 | 0.009 | 0.889 |
| ENSSMAG00000012669 | 0.889 | 0.031 | 1.000 |
| ENSSMAG00000010347 | 0.875 | 0.025 | 1.000 |
| ENSSMAG00000007906 | 0.873 | 0.050 | 1.000 |
| ENSSMAG00000020234 | 0.870 | 0.007 | 0.843 |
| ENSSMAG00000035605 | 0.868 | 0.049 | 1.000 |
| ENSSMAG00000008149 | 0.867 | 0.015 | 1.000 |
| ENSSMAG00000034534 | 0.847 | 0.014 | 1.000 |
| ENSSMAG00000015797 | 0.845 | 0.023 | 1.000 |
| ENSSMAG00000032784 | 0.845 | 0.038 | 1.000 |
| ENSSMAG00000010785 | 0.844 | 0.025 | 1.000 |
| ENSSMAG00000010248 | 0.839 | 0.017 | 1.000 |
| ENSSMAG00000023368 | 0.837 | 0.036 | 1.000 |
| ENSSMAG00000002190 | 0.836 | 0.049 | 1.000 |
| ENSSMAG00000034639 | 0.836 | 0.007 | 0.843 |
| ENSSMAG00000005992 | 0.836 | 0.002 | 0.532 |
| ENSSMAG00000035831 | 0.835 | 0.010 | 0.909 |
| ENSSMAG00000020374 | 0.835 | 0.011 | 0.980 |
| ENSSMAG00000005622 | 0.834 | 0.001 | 0.268 |
| ENSSMAG00000003487 | 0.833 | 0.043 | 1.000 |
| ENSSMAG00000003181 | 0.832 | 0.043 | 1.000 |
| ENSSMAG00000033383 | 0.826 | <0.001 | <0.001 |
| ENSSMAG00000019267 | 0.825 | 0.017 | 1.000 |
| ENSSMAG00000016911 | 0.825 | <0.001 | 0.156 |
| ENSSMAG00000014195 | 0.820 | 0.042 | 1.000 |
| ENSSMAG00000000909 | 0.818 | 0.026 | 1.000 |
| Novel.218 | 0.816 | 0.019 | 1.000 |
| ENSSMAG00000033287 | 0.812 | 0.003 | 0.620 |
| ENSSMAG00000006273 | 0.810 | 0.049 | 1.000 |
| ENSSMAG00000034016 | 0.794 | <0.001 | 0.131 |
| Novel.35 | 0.787 | 0.001 | 0.350 |
| ENSSMAG00000028380 | 0.785 | 0.001 | 0.382 |
| ENSSMAG00000028956 | 0.780 | 0.022 | 1.000 |
| ENSSMAG00000013156 | 0.774 | 0.005 | 0.728 |
| ENSSMAG00000019274 | 0.771 | 0.024 | 1.000 |
| ENSSMAG00000028301 | 0.770 | 0.009 | 0.891 |
| ENSSMAG00000037507 | 0.766 | 0.004 | 0.700 |
| ENSSMAG00000002793 | 0.765 | 0.002 | 0.532 |
| ENSSMAG00000016908 | 0.761 | 0.049 | 1.000 |
| ENSSMAG00000023490 | 0.760 | 0.003 | 0.620 |
| ENSSMAG00000018178 | 0.755 | 0.040 | 1.000 |
| ENSSMAG00000031645 | 0.754 | 0.030 | 1.000 |
| ENSSMAG00000020051 | 0.754 | 0.002 | 0.544 |
| ENSSMAG00000003566 | 0.753 | 0.001 | 0.271 |
| ENSSMAG00000001830 | 0.751 | 0.023 | 1.000 |
| ENSSMAG00000005563 | 0.747 | 0.027 | 1.000 |
| ENSSMAG00000000340 | 0.747 | 0.005 | 0.745 |
| ENSSMAG00000010919 | 0.744 | 0.002 | 0.499 |
| Novel.88 | 0.743 | 0.014 | 1.000 |
| ENSSMAG00000016189 | 0.739 | 0.023 | 1.000 |
| ENSSMAG00000017073 | 0.738 | 0.037 | 1.000 |
| ENSSMAG00000012744 | 0.734 | 0.040 | 1.000 |
| ENSSMAG00000032982 | 0.729 | <0.001 | 0.082 |
| ENSSMAG00000015791 | 0.729 | 0.031 | 1.000 |
| ENSSMAG00000011772 | 0.726 | 0.014 | 1.000 |
| ENSSMAG00000018801 | 0.726 | 0.050 | 1.000 |
| ENSSMAG00000002832 | 0.724 | 0.035 | 1.000 |
| ENSSMAG00000012956 | 0.713 | 0.003 | 0.611 |
| ENSSMAG00000022468 | 0.711 | <0.001 | 0.070 |
| ENSSMAG00000007778 | 0.711 | 0.032 | 1.000 |
| ENSSMAG00000012606 | 0.709 | 0.036 | 1.000 |
| ENSSMAG00000035702 | 0.709 | 0.013 | 0.993 |
| ENSSMAG00000019918 | 0.708 | 0.011 | 0.944 |
| ENSSMAG00000012861 | 0.702 | 0.005 | 0.734 |
| ENSSMAG00000016466 | 0.702 | 0.028 | 1.000 |
| ENSSMAG00000024801 | 0.698 | 0.048 | 1.000 |
| ENSSMAG00000008419 | 0.697 | 0.034 | 1.000 |
| ENSSMAG00000033634 | 0.695 | 0.026 | 1.000 |
| ENSSMAG00000019315 | 0.694 | 0.023 | 1.000 |
| ENSSMAG00000013974 | 0.687 | 0.001 | 0.300 |
| ENSSMAG00000005909 | 0.687 | 0.033 | 1.000 |
| ENSSMAG00000014029 | 0.685 | 0.002 | 0.470 |
| ENSSMAG00000009771 | 0.683 | 0.050 | 1.000 |
| ENSSMAG00000007796 | 0.683 | 0.016 | 1.000 |
| ENSSMAG00000023898 | 0.679 | 0.039 | 1.000 |
| ENSSMAG00000013885 | 0.679 | 0.040 | 1.000 |
| ENSSMAG00000037036 | 0.678 | 0.004 | 0.657 |
| ENSSMAG00000013034 | 0.671 | 0.040 | 1.000 |
| ENSSMAG00000024137 | 0.666 | 0.014 | 1.000 |
| ENSSMAG00000022952 | 0.665 | 0.018 | 1.000 |
| ENSSMAG00000019804 | 0.665 | 0.022 | 1.000 |
| ENSSMAG00000001298 | 0.654 | 0.009 | 0.889 |
| ENSSMAG00000011570 | 0.651 | 0.008 | 0.855 |
| Novel.233 | 0.651 | 0.041 | 1.000 |
| ENSSMAG00000017325 | 0.645 | 0.002 | 0.532 |
| ENSSMAG00000021417 | 0.645 | 0.028 | 1.000 |
| ENSSMAG00000021994 | 0.644 | 0.003 | 0.564 |
| ENSSMAG00000010650 | 0.642 | 0.016 | 1.000 |
| ENSSMAG00000011573 | 0.639 | 0.032 | 1.000 |
| ENSSMAG00000033535 | 0.639 | 0.041 | 1.000 |
| ENSSMAG00000017290 | 0.637 | 0.024 | 1.000 |
| ENSSMAG00000012809 | 0.636 | 0.027 | 1.000 |
| ENSSMAG00000019855 | 0.630 | 0.008 | 0.855 |
| ENSSMAG00000005308 | 0.628 | <0.001 | 0.183 |
| ENSSMAG00000021289 | 0.626 | 0.012 | 0.980 |
| ENSSMAG00000025947 | 0.624 | 0.050 | 1.000 |
| ENSSMAG00000034783 | 0.621 | 0.002 | 0.532 |
| ENSSMAG00000005260 | 0.620 | 0.003 | 0.606 |
| ENSSMAG00000029355 | 0.620 | 0.002 | 0.478 |
| ENSSMAG00000004239 | 0.620 | 0.020 | 1.000 |
| ENSSMAG00000012116 | 0.618 | 0.007 | 0.810 |
| ENSSMAG00000034406 | 0.618 | 0.018 | 1.000 |
| ENSSMAG00000013002 | 0.616 | 0.046 | 1.000 |
| ENSSMAG00000017138 | 0.614 | 0.004 | 0.692 |
| ENSSMAG00000004686 | 0.609 | 0.020 | 1.000 |
| ENSSMAG00000007895 | 0.609 | 0.019 | 1.000 |
| ENSSMAG00000000938 | 0.606 | 0.016 | 1.000 |
| ENSSMAG00000007850 | 0.605 | 0.019 | 1.000 |
| ENSSMAG00000006610 | 0.605 | 0.021 | 1.000 |
| ENSSMAG00000020780 | 0.601 | 0.026 | 1.000 |
| Novel.131 | 0.599 | 0.033 | 1.000 |
| ENSSMAG00000000789 | 0.597 | 0.036 | 1.000 |
| ENSSMAG00000013720 | 0.596 | 0.033 | 1.000 |
| ENSSMAG00000007885 | 0.595 | 0.012 | 0.984 |
| ENSSMAG00000002288 | 0.593 | 0.012 | 0.980 |
| ENSSMAG00000015994 | 0.593 | 0.009 | 0.889 |
| Novel.27 | 0.590 | 0.042 | 1.000 |
| ENSSMAG00000012335 | 0.589 | 0.007 | 0.835 |
| ENSSMAG00000006822 | 0.588 | 0.025 | 1.000 |
| ENSSMAG00000022743 | 0.588 | 0.040 | 1.000 |
| ENSSMAG00000013631 | 0.586 | 0.001 | 0.415 |
| ENSSMAG00000021767 | 0.586 | 0.046 | 1.000 |
| Novel.298 | 0.578 | 0.001 | 0.311 |
| ENSSMAG00000003358 | 0.578 | 0.043 | 1.000 |
| ENSSMAG00000032479 | 0.577 | 0.001 | 0.330 |
| ENSSMAG00000002531 | 0.576 | 0.036 | 1.000 |
| ENSSMAG00000006828 | 0.574 | 0.007 | 0.810 |
| ENSSMAG00000005715 | 0.573 | 0.018 | 1.000 |
| ENSSMAG00000031899 | 0.568 | 0.041 | 1.000 |
| ENSSMAG00000003012 | 0.564 | 0.047 | 1.000 |
| ENSSMAG00000013773 | 0.560 | 0.043 | 1.000 |
| ENSSMAG00000020719 | 0.557 | 0.003 | 0.580 |
| ENSSMAG00000033202 | 0.556 | 0.013 | 1.000 |
| ENSSMAG00000016682 | 0.556 | 0.040 | 1.000 |
| ENSSMAG00000008859 | 0.555 | 0.047 | 1.000 |
| ENSSMAG00000030809 | 0.555 | 0.005 | 0.728 |
| ENSSMAG00000017792 | 0.554 | 0.050 | 1.000 |
| ENSSMAG00000012576 | 0.554 | 0.004 | 0.690 |
| ENSSMAG00000006966 | 0.552 | 0.030 | 1.000 |
| ENSSMAG00000013553 | 0.551 | 0.025 | 1.000 |
| ENSSMAG00000019268 | 0.547 | 0.048 | 1.000 |
| Novel.351 | 0.547 | 0.023 | 1.000 |
| ENSSMAG00000011319 | 0.545 | 0.017 | 1.000 |
| ENSSMAG00000002983 | 0.545 | 0.004 | 0.672 |
| ENSSMAG00000015116 | 0.541 | 0.021 | 1.000 |
| ENSSMAG00000021005 | 0.541 | 0.002 | 0.543 |
| ENSSMAG00000004267 | 0.540 | 0.048 | 1.000 |
| ENSSMAG00000012302 | 0.540 | 0.020 | 1.000 |
| ENSSMAG00000027538 | 0.539 | 0.030 | 1.000 |
| ENSSMAG00000003248 | 0.538 | 0.010 | 0.904 |
| ENSSMAG00000006641 | 0.538 | 0.024 | 1.000 |
| ENSSMAG00000002972 | 0.536 | 0.047 | 1.000 |
| ENSSMAG00000017477 | 0.534 | 0.005 | 0.734 |
| ENSSMAG00000002692 | 0.532 | 0.022 | 1.000 |
| ENSSMAG00000018852 | 0.530 | 0.019 | 1.000 |
| ENSSMAG00000023098 | 0.530 | 0.045 | 1.000 |
| ENSSMAG00000013833 | 0.528 | 0.020 | 1.000 |
| ENSSMAG00000021203 | 0.527 | 0.016 | 1.000 |
| ENSSMAG00000035137 | 0.527 | 0.003 | 0.596 |
| ENSSMAG00000018258 | 0.524 | 0.050 | 1.000 |
| ENSSMAG00000015523 | 0.524 | 0.035 | 1.000 |
| ENSSMAG00000020676 | 0.523 | 0.024 | 1.000 |
| ENSSMAG00000006551 | 0.523 | 0.043 | 1.000 |
| ENSSMAG00000014106 | 0.520 | 0.023 | 1.000 |
| ENSSMAG00000006591 | 0.518 | 0.014 | 1.000 |
| ENSSMAG00000014554 | 0.518 | 0.047 | 1.000 |
| ENSSMAG00000005648 | 0.517 | 0.018 | 1.000 |
| Novel.222 | 0.514 | 0.050 | 1.000 |
| ENSSMAG00000021090 | 0.511 | 0.043 | 1.000 |
| ENSSMAG00000016753 | 0.509 | 0.036 | 1.000 |
| ENSSMAG00000000201 | 0.500 | 0.017 | 1.000 |
| ENSSMAG00000004771 | 0.498 | 0.007 | 0.826 |
| ENSSMAG00000005879 | 0.494 | 0.033 | 1.000 |
| ENSSMAG00000031531 | 0.494 | 0.003 | 0.604 |
| ENSSMAG00000012556 | 0.490 | 0.014 | 1.000 |
| ENSSMAG00000017797 | 0.487 | 0.006 | 0.770 |
| ENSSMAG00000033776 | 0.486 | 0.029 | 1.000 |
| ENSSMAG00000009114 | 0.483 | 0.007 | 0.843 |
| ENSSMAG00000000755 | 0.483 | 0.029 | 1.000 |
| ENSSMAG00000036172 | 0.481 | 0.041 | 1.000 |
| ENSSMAG00000022659 | 0.479 | 0.005 | 0.734 |
| ENSSMAG00000013445 | 0.479 | 0.035 | 1.000 |
| ENSSMAG00000008553 | 0.478 | 0.043 | 1.000 |
| ENSSMAG00000008487 | 0.478 | 0.041 | 1.000 |
| ENSSMAG00000019747 | 0.477 | 0.032 | 1.000 |
| ENSSMAG00000035989 | 0.477 | 0.008 | 0.855 |
| ENSSMAG00000016308 | 0.476 | 0.047 | 1.000 |
| ENSSMAG00000008854 | 0.476 | 0.032 | 1.000 |
| ENSSMAG00000036952 | 0.474 | 0.003 | 0.569 |
| ENSSMAG00000007038 | 0.468 | 0.007 | 0.843 |
| ENSSMAG00000000944 | 0.464 | 0.039 | 1.000 |
| ENSSMAG00000015704 | 0.463 | 0.035 | 1.000 |
| ENSSMAG00000020790 | 0.462 | 0.047 | 1.000 |
| ENSSMAG00000025230 | 0.461 | 0.049 | 1.000 |
| ENSSMAG00000013559 | 0.460 | 0.012 | 0.980 |
| ENSSMAG00000012770 | 0.459 | 0.036 | 1.000 |
| ENSSMAG00000012123 | 0.458 | 0.033 | 1.000 |
| Novel.82 | 0.456 | 0.031 | 1.000 |
| ENSSMAG00000030749 | 0.455 | 0.012 | 0.984 |
| ENSSMAG00000006168 | 0.455 | 0.035 | 1.000 |
| ENSSMAG00000019725 | 0.453 | 0.033 | 1.000 |
| ENSSMAG00000013018 | 0.450 | 0.043 | 1.000 |
| ENSSMAG00000015450 | 0.449 | 0.049 | 1.000 |
| ENSSMAG00000001251 | 0.445 | 0.045 | 1.000 |
| ENSSMAG00000037779 | 0.443 | 0.050 | 1.000 |
| ENSSMAG00000020804 | 0.441 | 0.033 | 1.000 |
| ENSSMAG00000034211 | 0.439 | 0.019 | 1.000 |
| ENSSMAG00000014941 | 0.437 | 0.041 | 1.000 |
| ENSSMAG00000030500 | 0.429 | 0.050 | 1.000 |
| ENSSMAG00000013437 | 0.421 | 0.035 | 1.000 |
| ENSSMAG00000005041 | 0.419 | 0.009 | 0.889 |
| ENSSMAG00000030557 | 0.419 | 0.019 | 1.000 |
| Novel.372 | 0.416 | 0.047 | 1.000 |
| ENSSMAG00000027450 | 0.416 | 0.038 | 1.000 |
| ENSSMAG00000007610 | 0.415 | 0.022 | 1.000 |
| ENSSMAG00000015774 | 0.413 | 0.015 | 1.000 |
| ENSSMAG00000014738 | 0.410 | 0.029 | 1.000 |
| ENSSMAG00000021956 | 0.410 | 0.028 | 1.000 |
| ENSSMAG00000037283 | 0.406 | 0.040 | 1.000 |
| ENSSMAG00000002821 | 0.400 | 0.015 | 1.000 |
| ENSSMAG00000000015 | 0.398 | 0.033 | 1.000 |
| ENSSMAG00000017791 | 0.395 | 0.048 | 1.000 |
| ENSSMAG00000003905 | 0.395 | 0.019 | 1.000 |
| ENSSMAG00000000370 | 0.393 | 0.012 | 0.980 |
| ENSSMAG00000017703 | 0.387 | 0.050 | 1.000 |
| ENSSMAG00000010411 | 0.380 | 0.040 | 1.000 |
| ENSSMAG00000022350 | 0.379 | 0.047 | 1.000 |
| ENSSMAG00000001998 | 0.378 | 0.035 | 1.000 |
| ENSSMAG00000000381 | 0.377 | 0.035 | 1.000 |
| ENSSMAG00000012137 | 0.375 | 0.047 | 1.000 |
| ENSSMAG00000015504 | 0.372 | 0.040 | 1.000 |
| ENSSMAG00000009595 | 0.371 | 0.020 | 1.000 |
| ENSSMAG00000016341 | 0.371 | 0.045 | 1.000 |
| ENSSMAG00000009398 | 0.370 | 0.026 | 1.000 |
| ENSSMAG00000016699 | 0.367 | 0.046 | 1.000 |
| ENSSMAG00000015064 | 0.365 | 0.048 | 1.000 |
| ENSSMAG00000016533 | 0.361 | 0.042 | 1.000 |
| ENSSMAG00000005307 | 0.361 | 0.026 | 1.000 |
| ENSSMAG00000035890 | 0.360 | 0.043 | 1.000 |
| ENSSMAG00000004547 | 0.319 | 0.049 | 1.000 |
| ENSSMAG00000006504 | -0.307 | 0.039 | 1.000 |
| ENSSMAG00000002292 | -0.314 | 0.036 | 1.000 |
| ENSSMAG00000006080 | -0.315 | 0.047 | 1.000 |
| ENSSMAG00000016964 | -0.325 | 0.026 | 1.000 |
| ENSSMAG00000013248 | -0.328 | 0.047 | 1.000 |
| ENSSMAG00000027991 | -0.329 | 0.031 | 1.000 |
| ENSSMAG00000003150 | -0.332 | 0.036 | 1.000 |
| ENSSMAG00000006794 | -0.333 | 0.045 | 1.000 |
| ENSSMAG00000017784 | -0.339 | 0.049 | 1.000 |
| ENSSMAG00000034648 | -0.344 | 0.049 | 1.000 |
| ENSSMAG00000002865 | -0.344 | 0.041 | 1.000 |
| ENSSMAG00000003390 | -0.345 | 0.041 | 1.000 |
| ENSSMAG00000021443 | -0.346 | 0.033 | 1.000 |
| ENSSMAG00000011486 | -0.347 | 0.024 | 1.000 |
| ENSSMAG00000005758 | -0.349 | 0.048 | 1.000 |
| ENSSMAG00000021283 | -0.350 | 0.031 | 1.000 |
| ENSSMAG00000010208 | -0.360 | 0.045 | 1.000 |
| ENSSMAG00000026407 | -0.362 | 0.044 | 1.000 |
| ENSSMAG00000006565 | -0.362 | 0.046 | 1.000 |
| ENSSMAG00000017016 | -0.366 | 0.010 | 0.909 |
| ENSSMAG00000005840 | -0.369 | 0.029 | 1.000 |
| ENSSMAG00000012418 | -0.374 | 0.049 | 1.000 |
| ENSSMAG00000031093 | -0.374 | 0.028 | 1.000 |
| ENSSMAG00000012603 | -0.377 | 0.034 | 1.000 |
| ENSSMAG00000017486 | -0.378 | 0.025 | 1.000 |
| ENSSMAG00000038023 | -0.380 | 0.024 | 1.000 |
| ENSSMAG00000011150 | -0.381 | 0.020 | 1.000 |
| ENSSMAG00000002358 | -0.384 | 0.021 | 1.000 |
| ENSSMAG00000030149 | -0.388 | 0.047 | 1.000 |
| ENSSMAG00000010334 | -0.389 | 0.031 | 1.000 |
| ENSSMAG00000004637 | -0.391 | 0.013 | 0.993 |
| ENSSMAG00000007688 | -0.393 | 0.023 | 1.000 |
| ENSSMAG00000004535 | -0.393 | 0.016 | 1.000 |
| ENSSMAG00000020610 | -0.393 | 0.037 | 1.000 |
| ENSSMAG00000021101 | -0.394 | 0.015 | 1.000 |
| ENSSMAG00000004362 | -0.398 | 0.023 | 1.000 |
| ENSSMAG00000029617 | -0.399 | 0.032 | 1.000 |
| ENSSMAG00000016876 | -0.399 | 0.049 | 1.000 |
| ENSSMAG00000016886 | -0.399 | 0.026 | 1.000 |
| ENSSMAG00000021079 | -0.400 | 0.044 | 1.000 |
| ENSSMAG00000002290 | -0.401 | 0.025 | 1.000 |
| ENSSMAG00000009930 | -0.401 | 0.021 | 1.000 |
| ENSSMAG00000026125 | -0.401 | 0.010 | 0.904 |
| ENSSMAG00000008081 | -0.403 | 0.019 | 1.000 |
| ENSSMAG00000012662 | -0.403 | 0.035 | 1.000 |
| ENSSMAG00000003375 | -0.407 | 0.034 | 1.000 |
| ENSSMAG00000008823 | -0.408 | 0.014 | 1.000 |
| ENSSMAG00000006381 | -0.408 | 0.020 | 1.000 |
| ENSSMAG00000002730 | -0.410 | 0.024 | 1.000 |
| ENSSMAG00000012166 | -0.411 | 0.048 | 1.000 |
| ENSSMAG00000019992 | -0.412 | 0.021 | 1.000 |
| ENSSMAG00000015912 | -0.414 | 0.037 | 1.000 |
| ENSSMAG00000006347 | -0.415 | 0.023 | 1.000 |
| ENSSMAG00000002272 | -0.417 | 0.030 | 1.000 |
| ENSSMAG00000020457 | -0.421 | 0.024 | 1.000 |
| ENSSMAG00000015866 | -0.423 | 0.019 | 1.000 |
| ENSSMAG00000005011 | -0.423 | 0.031 | 1.000 |
| ENSSMAG00000007678 | -0.424 | 0.025 | 1.000 |
| ENSSMAG00000003602 | -0.424 | 0.021 | 1.000 |
| ENSSMAG00000013681 | -0.425 | 0.050 | 1.000 |
| ENSSMAG00000021679 | -0.426 | 0.044 | 1.000 |
| ENSSMAG00000011720 | -0.429 | 0.028 | 1.000 |
| ENSSMAG00000015973 | -0.430 | 0.022 | 1.000 |
| ENSSMAG00000011675 | -0.432 | 0.031 | 1.000 |
| ENSSMAG00000018231 | -0.433 | 0.012 | 0.980 |
| ENSSMAG00000017107 | -0.433 | 0.037 | 1.000 |
| ENSSMAG00000006717 | -0.434 | 0.017 | 1.000 |
| ENSSMAG00000002687 | -0.435 | 0.048 | 1.000 |
| ENSSMAG00000011351 | -0.435 | 0.022 | 1.000 |
| ENSSMAG00000020486 | -0.435 | 0.028 | 1.000 |
| ENSSMAG00000012180 | -0.436 | 0.022 | 1.000 |
| ENSSMAG00000004608 | -0.438 | 0.016 | 1.000 |
| ENSSMAG00000012981 | -0.440 | 0.036 | 1.000 |
| ENSSMAG00000019090 | -0.440 | 0.014 | 1.000 |
| ENSSMAG00000017974 | -0.441 | 0.023 | 1.000 |
| ENSSMAG00000003015 | -0.442 | 0.010 | 0.904 |
| ENSSMAG00000006799 | -0.444 | 0.030 | 1.000 |
| ENSSMAG00000028932 | -0.446 | 0.017 | 1.000 |
| ENSSMAG00000021033 | -0.446 | 0.020 | 1.000 |
| ENSSMAG00000001645 | -0.447 | 0.005 | 0.728 |
| ENSSMAG00000007111 | -0.452 | 0.014 | 1.000 |
| ENSSMAG00000031819 | -0.454 | 0.029 | 1.000 |
| ENSSMAG00000007926 | -0.455 | 0.043 | 1.000 |
| ENSSMAG00000025258 | -0.459 | 0.023 | 1.000 |
| ENSSMAG00000015159 | -0.460 | 0.021 | 1.000 |
| ENSSMAG00000012501 | -0.462 | 0.045 | 1.000 |
| ENSSMAG00000014235 | -0.463 | 0.038 | 1.000 |
| ENSSMAG00000006523 | -0.463 | 0.032 | 1.000 |
| ENSSMAG00000032655 | -0.464 | 0.044 | 1.000 |
| ENSSMAG00000000993 | -0.465 | 0.045 | 1.000 |
| ENSSMAG00000013835 | -0.465 | 0.007 | 0.837 |
| ENSSMAG00000021078 | -0.466 | 0.020 | 1.000 |
| ENSSMAG00000012111 | -0.466 | 0.015 | 1.000 |
| ENSSMAG00000008111 | -0.469 | 0.009 | 0.889 |
| ENSSMAG00000006788 | -0.469 | 0.008 | 0.855 |
| ENSSMAG00000001489 | -0.471 | 0.020 | 1.000 |
| ENSSMAG00000023286 | -0.475 | 0.030 | 1.000 |
| ENSSMAG00000000398 | -0.476 | 0.026 | 1.000 |
| ENSSMAG00000034807 | -0.476 | 0.029 | 1.000 |
| ENSSMAG00000009550 | -0.477 | 0.035 | 1.000 |
| ENSSMAG00000009823 | -0.477 | 0.045 | 1.000 |
| ENSSMAG00000019403 | -0.478 | 0.009 | 0.889 |
| ENSSMAG00000035373 | -0.479 | 0.015 | 1.000 |
| ENSSMAG00000013412 | -0.480 | 0.042 | 1.000 |
| ENSSMAG00000017556 | -0.482 | 0.039 | 1.000 |
| ENSSMAG00000034039 | -0.483 | 0.001 | 0.387 |
| ENSSMAG00000033887 | -0.488 | 0.008 | 0.843 |
| ENSSMAG00000009445 | -0.488 | 0.011 | 0.952 |
| ENSSMAG00000006390 | -0.489 | 0.016 | 1.000 |
| ENSSMAG00000007845 | -0.490 | 0.014 | 1.000 |
| ENSSMAG00000016038 | -0.491 | 0.026 | 1.000 |
| ENSSMAG00000005398 | -0.491 | 0.042 | 1.000 |
| ENSSMAG00000034624 | -0.492 | 0.043 | 1.000 |
| ENSSMAG00000035315 | -0.493 | 0.011 | 0.952 |
| Novel.174 | -0.494 | 0.001 | 0.311 |
| ENSSMAG00000011666 | -0.494 | 0.030 | 1.000 |
| ENSSMAG00000010358 | -0.495 | 0.020 | 1.000 |
| ENSSMAG00000005258 | -0.496 | 0.002 | 0.533 |
| ENSSMAG00000013476 | -0.498 | 0.043 | 1.000 |
| ENSSMAG00000019164 | -0.498 | 0.021 | 1.000 |
| ENSSMAG00000016458 | -0.499 | 0.021 | 1.000 |
| ENSSMAG00000009453 | -0.500 | 0.036 | 1.000 |
| Novel.368 | -0.501 | 0.028 | 1.000 |
| ENSSMAG00000010099 | -0.503 | 0.025 | 1.000 |
| ENSSMAG00000004772 | -0.503 | 0.015 | 1.000 |
| ENSSMAG00000025811 | -0.503 | 0.010 | 0.937 |
| ENSSMAG00000012101 | -0.504 | 0.045 | 1.000 |
| ENSSMAG00000010832 | -0.506 | 0.020 | 1.000 |
| ENSSMAG00000020769 | -0.507 | 0.049 | 1.000 |
| ENSSMAG00000001365 | -0.507 | 0.008 | 0.855 |
| ENSSMAG00000021221 | -0.508 | 0.046 | 1.000 |
| ENSSMAG00000014755 | -0.509 | 0.025 | 1.000 |
| ENSSMAG00000017920 | -0.512 | 0.018 | 1.000 |
| ENSSMAG00000016480 | -0.514 | 0.014 | 1.000 |
| ENSSMAG00000014422 | -0.514 | 0.001 | 0.415 |
| ENSSMAG00000021122 | -0.517 | 0.043 | 1.000 |
| ENSSMAG00000014869 | -0.518 | 0.002 | 0.532 |
| ENSSMAG00000006765 | -0.518 | 0.008 | 0.855 |
| ENSSMAG00000019916 | -0.520 | 0.019 | 1.000 |
| ENSSMAG00000007308 | -0.520 | 0.031 | 1.000 |
| ENSSMAG00000034191 | -0.522 | 0.022 | 1.000 |
| ENSSMAG00000002760 | -0.523 | 0.027 | 1.000 |
| ENSSMAG00000000061 | -0.525 | 0.041 | 1.000 |
| ENSSMAG00000021044 | -0.526 | 0.024 | 1.000 |
| ENSSMAG00000010326 | -0.527 | 0.028 | 1.000 |
| ENSSMAG00000020210 | -0.530 | 0.033 | 1.000 |
| ENSSMAG00000020080 | -0.533 | 0.025 | 1.000 |
| ENSSMAG00000010712 | -0.533 | 0.035 | 1.000 |
| ENSSMAG00000023865 | -0.534 | 0.042 | 1.000 |
| ENSSMAG00000007177 | -0.538 | 0.023 | 1.000 |
| ENSSMAG00000000471 | -0.540 | 0.007 | 0.843 |
| ENSSMAG00000001543 | -0.541 | 0.036 | 1.000 |
| ENSSMAG00000016651 | -0.541 | 0.012 | 0.980 |
| ENSSMAG00000012092 | -0.542 | 0.048 | 1.000 |
| ENSSMAG00000011844 | -0.543 | 0.007 | 0.843 |
| ENSSMAG00000033129 | -0.548 | 0.036 | 1.000 |
| ENSSMAG00000036742 | -0.550 | 0.021 | 1.000 |
| ENSSMAG00000018878 | -0.555 | 0.037 | 1.000 |
| ENSSMAG00000001394 | -0.556 | 0.015 | 1.000 |
| ENSSMAG00000011332 | -0.556 | 0.028 | 1.000 |
| ENSSMAG00000007343 | -0.557 | 0.024 | 1.000 |
| ENSSMAG00000037341 | -0.558 | 0.004 | 0.661 |
| ENSSMAG00000006418 | -0.559 | 0.046 | 1.000 |
| ENSSMAG00000019938 | -0.560 | 0.035 | 1.000 |
| ENSSMAG00000004775 | -0.561 | 0.010 | 0.904 |
| ENSSMAG00000016084 | -0.565 | 0.034 | 1.000 |
| ENSSMAG00000012753 | -0.573 | 0.020 | 1.000 |
| ENSSMAG00000030523 | -0.577 | 0.005 | 0.728 |
| ENSSMAG00000009572 | -0.577 | 0.017 | 1.000 |
| ENSSMAG00000025401 | -0.581 | 0.035 | 1.000 |
| ENSSMAG00000004722 | -0.582 | <0.001 | 0.118 |
| ENSSMAG00000031390 | -0.583 | 0.034 | 1.000 |
| ENSSMAG00000006598 | -0.584 | 0.004 | 0.690 |
| ENSSMAG00000006350 | -0.585 | 0.015 | 1.000 |
| ENSSMAG00000003111 | -0.585 | 0.005 | 0.734 |
| ENSSMAG00000034900 | -0.587 | 0.024 | 1.000 |
| ENSSMAG00000037977 | -0.587 | 0.015 | 1.000 |
| ENSSMAG00000016011 | -0.587 | 0.046 | 1.000 |
| ENSSMAG00000010961 | -0.587 | 0.010 | 0.904 |
| ENSSMAG00000004087 | -0.588 | 0.042 | 1.000 |
| ENSSMAG00000013698 | -0.588 | 0.013 | 1.000 |
| ENSSMAG00000006175 | -0.591 | 0.018 | 1.000 |
| ENSSMAG00000012372 | -0.594 | 0.035 | 1.000 |
| ENSSMAG00000005239 | -0.594 | 0.006 | 0.770 |
| ENSSMAG00000030174 | -0.594 | 0.002 | 0.493 |
| ENSSMAG00000001863 | -0.604 | 0.003 | 0.611 |
| ENSSMAG00000023453 | -0.605 | 0.012 | 0.980 |
| ENSSMAG00000017607 | -0.607 | 0.020 | 1.000 |
| ENSSMAG00000014181 | -0.607 | 0.005 | 0.728 |
| ENSSMAG00000020324 | -0.609 | 0.002 | 0.493 |
| ENSSMAG00000010634 | -0.614 | 0.017 | 1.000 |
| ENSSMAG00000016265 | -0.614 | 0.041 | 1.000 |
| ENSSMAG00000008949 | -0.622 | 0.033 | 1.000 |
| ENSSMAG00000007877 | -0.629 | 0.004 | 0.659 |
| ENSSMAG00000003979 | -0.630 | 0.011 | 0.944 |
| ENSSMAG00000002910 | -0.631 | <0.001 | 0.183 |
| ENSSMAG00000017123 | -0.633 | 0.031 | 1.000 |
| ENSSMAG00000001802 | -0.633 | 0.006 | 0.760 |
| ENSSMAG00000002256 | -0.637 | 0.019 | 1.000 |
| ENSSMAG00000014018 | -0.648 | 0.006 | 0.770 |
| ENSSMAG00000002042 | -0.648 | 0.029 | 1.000 |
| ENSSMAG00000037313 | -0.651 | 0.014 | 1.000 |
| ENSSMAG00000001117 | -0.654 | 0.013 | 0.993 |
| ENSSMAG00000004695 | -0.656 | 0.028 | 1.000 |
| ENSSMAG00000018229 | -0.656 | <0.001 | 0.070 |
| ENSSMAG00000017670 | -0.659 | 0.023 | 1.000 |
| ENSSMAG00000009733 | -0.668 | 0.040 | 1.000 |
| ENSSMAG00000027097 | -0.670 | 0.026 | 1.000 |
| ENSSMAG00000007273 | -0.672 | 0.006 | 0.778 |
| ENSSMAG00000018732 | -0.672 | <0.001 | 0.083 |
| ENSSMAG00000025915 | -0.674 | 0.046 | 1.000 |
| ENSSMAG00000037037 | -0.679 | 0.014 | 1.000 |
| Novel.248 | -0.681 | 0.025 | 1.000 |
| ENSSMAG00000000659 | -0.685 | 0.025 | 1.000 |
| ENSSMAG00000026164 | -0.686 | <0.001 | 0.047 |
| ENSSMAG00000017571 | -0.692 | 0.049 | 1.000 |
| ENSSMAG00000031209 | -0.694 | 0.028 | 1.000 |
| ENSSMAG00000005592 | -0.696 | 0.023 | 1.000 |
| ENSSMAG00000007239 | -0.697 | 0.020 | 1.000 |
| ENSSMAG00000005327 | -0.698 | 0.026 | 1.000 |
| ENSSMAG00000021210 | -0.698 | 0.047 | 1.000 |
| ENSSMAG00000003393 | -0.699 | 0.020 | 1.000 |
| ENSSMAG00000011088 | -0.699 | 0.004 | 0.675 |
| ENSSMAG00000000822 | -0.703 | 0.017 | 1.000 |
| ENSSMAG00000005808 | -0.703 | 0.012 | 0.980 |
| ENSSMAG00000018781 | -0.704 | <0.001 | 0.131 |
| ENSSMAG00000003109 | -0.712 | 0.021 | 1.000 |
| ENSSMAG00000013705 | -0.714 | 0.028 | 1.000 |
| ENSSMAG00000035413 | -0.716 | 0.045 | 1.000 |
| ENSSMAG00000003871 | -0.716 | 0.027 | 1.000 |
| ENSSMAG00000002252 | -0.718 | 0.014 | 1.000 |
| ENSSMAG00000007814 | -0.719 | 0.008 | 0.855 |
| ENSSMAG00000006520 | -0.720 | 0.023 | 1.000 |
| Novel.304 | -0.721 | 0.045 | 1.000 |
| ENSSMAG00000007363 | -0.724 | 0.005 | 0.734 |
| ENSSMAG00000005705 | -0.724 | 0.047 | 1.000 |
| ENSSMAG00000018359 | -0.733 | 0.028 | 1.000 |
| ENSSMAG00000003130 | -0.736 | 0.034 | 1.000 |
| ENSSMAG00000013821 | -0.741 | 0.039 | 1.000 |
| ENSSMAG00000014558 | -0.741 | 0.003 | 0.564 |
| ENSSMAG00000008177 | -0.744 | 0.033 | 1.000 |
| ENSSMAG00000001686 | -0.745 | 0.030 | 1.000 |
| ENSSMAG00000018449 | -0.745 | 0.009 | 0.889 |
| ENSSMAG00000002347 | -0.758 | 0.035 | 1.000 |
| ENSSMAG00000036836 | -0.761 | 0.008 | 0.855 |
| ENSSMAG00000013511 | -0.773 | <0.001 | 0.118 |
| ENSSMAG00000019411 | -0.779 | 0.040 | 1.000 |
| ENSSMAG00000005507 | -0.780 | 0.025 | 1.000 |
| ENSSMAG00000006599 | -0.781 | 0.013 | 0.997 |
| ENSSMAG00000002606 | -0.787 | 0.039 | 1.000 |
| ENSSMAG00000018585 | -0.795 | 0.006 | 0.787 |
| ENSSMAG00000013227 | -0.797 | 0.039 | 1.000 |
| ENSSMAG00000009811 | -0.800 | 0.030 | 1.000 |
| ENSSMAG00000006898 | -0.804 | 0.027 | 1.000 |
| ENSSMAG00000011822 | -0.810 | 0.025 | 1.000 |
| ENSSMAG00000010864 | -0.820 | 0.024 | 1.000 |
| ENSSMAG00000012739 | -0.825 | 0.034 | 1.000 |
| ENSSMAG00000008891 | -0.830 | 0.042 | 1.000 |
| ENSSMAG00000006213 | -0.832 | 0.016 | 1.000 |
| ENSSMAG00000009313 | -0.837 | 0.030 | 1.000 |
| ENSSMAG00000012474 | -0.838 | 0.037 | 1.000 |
| ENSSMAG00000011611 | -0.842 | 0.018 | 1.000 |
| ENSSMAG00000015460 | -0.850 | 0.023 | 1.000 |
| ENSSMAG00000008146 | -0.851 | <0.001 | 0.007 |
| ENSSMAG00000000054 | -0.851 | 0.038 | 1.000 |
| ENSSMAG00000025898 | -0.862 | 0.045 | 1.000 |
| ENSSMAG00000011930 | -0.867 | 0.010 | 0.906 |
| ENSSMAG00000003144 | -0.877 | 0.027 | 1.000 |
| ENSSMAG00000031157 | -0.891 | 0.024 | 1.000 |
| ENSSMAG00000017480 | -0.899 | <0.001 | <0.001 |
| ENSSMAG00000013340 | -0.903 | 0.012 | 0.980 |
| ENSSMAG00000026109 | -0.911 | 0.041 | 1.000 |
| ENSSMAG00000006581 | -0.914 | 0.040 | 1.000 |
| ENSSMAG00000002004 | -0.915 | 0.021 | 1.000 |
| ENSSMAG00000015101 | -0.919 | 0.001 | 0.241 |
| ENSSMAG00000023910 | -0.925 | 0.018 | 1.000 |
| ENSSMAG00000021085 | -0.927 | 0.036 | 1.000 |
| ENSSMAG00000013783 | -0.929 | 0.026 | 1.000 |
| ENSSMAG00000012730 | -0.936 | 0.002 | 0.532 |
| ENSSMAG00000003219 | -0.939 | 0.009 | 0.889 |
| ENSSMAG00000000382 | -0.941 | 0.014 | 1.000 |
| ENSSMAG00000021701 | -0.945 | 0.022 | 1.000 |
| ENSSMAG00000010177 | -0.948 | 0.017 | 1.000 |
| Novel.102 | -0.961 | 0.039 | 1.000 |
| ENSSMAG00000011420 | -0.980 | 0.020 | 1.000 |
| ENSSMAG00000005145 | -0.981 | 0.043 | 1.000 |
| ENSSMAG00000008395 | -0.982 | <0.001 | 0.008 |
| ENSSMAG00000017871 | -0.994 | 0.007 | 0.810 |
| ENSSMAG00000034931 | -0.997 | 0.005 | 0.728 |
| ENSSMAG00000005080 | -0.997 | 0.033 | 1.000 |
| Novel.114 | -1.008 | 0.009 | 0.889 |
| ENSSMAG00000029421 | -1.011 | 0.009 | 0.889 |
| ENSSMAG00000004300 | -1.026 | 0.028 | 1.000 |
| ENSSMAG00000020123 | -1.054 | 0.003 | 0.611 |
| ENSSMAG00000004319 | -1.058 | 0.038 | 1.000 |
| ENSSMAG00000017159 | -1.062 | 0.008 | 0.855 |
| ENSSMAG00000007391 | -1.071 | 0.001 | 0.349 |
| Novel.267 | -1.082 | 0.042 | 1.000 |
| ENSSMAG00000006893 | -1.102 | <0.001 | 0.015 |
| ENSSMAG00000015329 | -1.111 | 0.033 | 1.000 |
| ENSSMAG00000019866 | -1.127 | <0.001 | 0.002 |
| ENSSMAG00000010416 | -1.129 | <0.001 | 0.029 |
| ENSSMAG00000033956 | -1.129 | 0.047 | 1.000 |
| ENSSMAG00000020897 | -1.138 | 0.003 | 0.611 |
| ENSSMAG00000016392 | -1.141 | 0.012 | 0.980 |
| ENSSMAG00000004446 | -1.168 | 0.009 | 0.891 |
| ENSSMAG00000016467 | -1.180 | 0.003 | 0.611 |
| ENSSMAG00000020003 | -1.188 | 0.026 | 1.000 |
| ENSSMAG00000007346 | -1.207 | 0.005 | 0.734 |
| ENSSMAG00000003647 | -1.224 | 0.017 | 1.000 |
| ENSSMAG00000010464 | -1.235 | 0.034 | 1.000 |
| ENSSMAG00000016851 | -1.242 | 0.039 | 1.000 |
| ENSSMAG00000027307 | -1.248 | 0.033 | 1.000 |
| ENSSMAG00000003459 | -1.248 | 0.047 | 1.000 |
| ENSSMAG00000002034 | -1.274 | 0.011 | 0.980 |
| ENSSMAG00000001674 | -1.283 | 0.022 | 1.000 |
| ENSSMAG00000007216 | -1.287 | 0.033 | 1.000 |
| ENSSMAG00000018846 | -1.290 | 0.047 | 1.000 |
| ENSSMAG00000000168 | -1.301 | 0.028 | 1.000 |
| ENSSMAG00000002176 | -1.305 | 0.002 | 0.532 |
| ENSSMAG00000004611 | -1.345 | 0.035 | 1.000 |
| ENSSMAG00000001849 | -1.352 | 0.041 | 1.000 |
| Novel.268 | -1.361 | 0.021 | 1.000 |
| ENSSMAG00000028217 | -1.416 | 0.024 | 1.000 |
| ENSSMAG00000029159 | -1.418 | 0.020 | 1.000 |
| ENSSMAG00000017923 | -1.433 | 0.049 | 1.000 |
| Novel.336 | -1.459 | 0.003 | 0.611 |
| ENSSMAG00000022169 | -1.462 | 0.038 | 1.000 |
| ENSSMAG00000008701 | -1.466 | 0.002 | 0.532 |
| ENSSMAG00000009374 | -1.489 | 0.018 | 1.000 |
| ENSSMAG00000018373 | -1.501 | 0.003 | 0.620 |
| ENSSMAG00000026542 | -1.501 | 0.023 | 1.000 |
| ENSSMAG00000004126 | -1.505 | 0.010 | 0.906 |
| ENSSMAG00000017004 | -1.512 | 0.003 | 0.606 |
| ENSSMAG00000004208 | -1.555 | 0.040 | 1.000 |
| ENSSMAG00000037225 | -1.591 | 0.002 | 0.457 |
| ENSSMAG00000036316 | -1.621 | 0.021 | 1.000 |
| ENSSMAG00000019068 | -1.745 | 0.021 | 1.000 |
| ENSSMAG00000034886 | -1.805 | 0.046 | 1.000 |
| ENSSMAG00000024591 | -1.857 | 0.032 | 1.000 |
| ENSSMAG00000001252 | -1.896 | 0.023 | 1.000 |
| ENSSMAG00000009900 | -1.917 | 0.040 | 1.000 |
| ENSSMAG00000002640 | -1.976 | 0.026 | 1.000 |
| Novel.278 | -1.988 | 0.042 | 1.000 |
| ENSSMAG00000015109 | -2.042 | 0.002 | 0.532 |
| ENSSMAG00000016763 | -2.232 | 0.028 | 1.000 |
| Novel.250 | -2.249 | 0.027 | 1.000 |
| ENSSMAG00000017274 | -2.296 | 0.023 | 1.000 |
| ENSSMAG00000006055 | -2.404 | 0.050 | 1.000 |
| ENSSMAG00000009625 | -2.598 | 0.028 | 1.000 |
| ENSSMAG00000011679 | -2.881 | 0.036 | 1.000 |
| ENSSMAG00000000005 | -3.548 | 0.045 | 1.000 |
| ENSSMAG00000019698 | -3.670 | 0.036 | 1.000 |
| ENSSMAG00000018381 | -3.779 | 0.033 | 1.000 |
| ENSSMAG00000009450 | -3.824 | 0.023 | 1.000 |
| ENSSMAG00000021388 | -3.928 | 0.044 | 1.000 |
| ENSSMAG00000019887 | -3.980 | 0.018 | 1.000 |
| ENSSMAG00000024679 | -4.179 | 0.034 | 1.000 |
| Novel.261 | -4.215 | 0.021 | 1.000 |
| ENSSMAG00000036949 | -4.641 | <0.001 | 0.083 |
| ENSSMAG00000012312 | -4.669 | 0.025 | 1.000 |
| ENSSMAG00000001792 | -4.847 | 0.011 | 0.948 |
| ENSSMAG00000001551 | -4.864 | 0.023 | 1.000 |
| ENSSMAG00000028205 | -5.059 | 0.007 | 0.810 |
| ENSSMAG00000032870 | -5.120 | 0.008 | 0.855 |
| ENSSMAG00000020172 | -5.457 | 0.001 | 0.382 |
| ENSSMAG00000037172 | -7.103 | 0.013 | 0.993 |

**Table S6** Detailed information (initial fish body weight, water temperature, and fasting or refeeding duration) of research examples mentioned in the manuscript.

| Common name | Scientific names | IBW, g | WT, °C | Feeding cessation WT, °C | Fasting duration, d | Fasting ADD^1^, °C·d | Refeeding duration, d | Refeeding ADD, °C·d | CG | References |
| --- | --- | --- | --- | --- | --- | --- | --- | --- | --- | --- |
| Turbot | *Scophthalmus maximus* | 5.40 | 18.0 | 8.00 | 3 | 30 | 60 | 600 | No CG | The present study |
|  |  |  |  |  | 6 | 60 |  |  |  |  |
|  |  |  |  |  | 9 | 90 |  |  |  |  |
|  |  |  |  |  | 12 | 120 |  |  |  |  |
| Yellowfin seabream | *Acanthopagrus latus* | 2.40 | 27.5 | 10.0 | 4 | 70 | 16 | 280 | Partial | Tamadoni et al., 2020 |
|  |  |  |  |  | 8 | 140 | 32 | 550 | Partial |  |
| Yellowfin seabream | *Acanthopagrus latus* | 0.80 | 27.2 | 10.0 | 30 | 516 | 30 | 516 | Partial | Mozanzadeh et al., 2020 |
| Longsnout catfish | *Leiocassis longirostris* | 13.1 | 28.0 | 10.0 | 7 | 126 | 28 | 504 | Complete | Zhu et al., 2005 |
| Yellowfin seabream | *Acanthopagrus latus* | 4.30 | 29.2 | 10.0 | 7 | 134 | 35 | 672 | Partial | Mozanzadeh et al., 2021 |
|  |  |  |  |  | 14 | 269 |  |  | Partial |  |
|  |  |  |  |  | 21 | 403 |  |  | Partial |  |
| Korean rockfish | *Sebastes schlegelii* | 1.43 | 25.5 | 5.00 | 10 | 205 | 35 | 718 | Partial | Oh et al., 2008 |
|  |  |  |  |  | 14 | 287 |  |  | Partial |  |
| Nile tilapia | *Oreochromis niloticus* | 9.34 | 28.3 | 12.0 | 5 | 82 | 7 | 114 | No CG | Mishra et al., 2025 |
|  |  |  |  |  | 7 | 114 |  |  | No CG |  |
| Tongue sole | *Cynoglossus semilaevis* | 2.79 | 22.0 | 10.0 | 4 | 48.0 | 60 | 720 | Complete | Tian et al., 2010 |
|  |  |  |  |  | 8 | 96.0 |  |  | Partial |  |
|  |  |  |  |  | 16 | 192 |  |  | Partial |  |
|  |  |  |  |  | 32 | 384 |  |  | Partial |  |
| Rohu | *Labeo rohita* | 3.75 | 28.2 | 14.0 | 7 | 99.4 | 35 | 497 | Partial | Yengkokpam et al., 2014 |
|  |  |  |  |  | 14 | 199 |  |  | partial |  |
| Nile tilapia | *Oreochromis niloticus* | 8.90 | 25.0 | 12.0 | 7 | 91.0 | 84 | 1092 | Complete | Abdel-Tawwab et al., 2006 |
|  |  |  |  |  | 14 | 182 | 77 | 1001 | No CG |  |
|  |  |  |  |  | 21 | 273 | 70 | 910 | No CG |  |
|  |  |  |  |  | 28 | 364 | 63 | 819 | No CG |  |
| Siberian sturgeon | *Acipenser baerii* | 46.5 | 17.2 | 3.00 | 21 | 298 | 28 | 398 | Partial | Ashouri et al., 2020 |
| Sobaity bream | *Sparidentex hasta* | 33.3 | 18.6 | 15.0 | 60 | 216 | 120 | 432 | Partial | Mozanzadeh et al., 2017 |
| Striped knifejaw | *Oplegnathus fasciatus* | 56.0 | 25.1 | 12.0 | 14 | 183 | 42 | 550 | Complete | Oh and Park, 2019 |
| Red seabream | *Pagrus major* | 77.1 | 25.5 | 10.0 | 7 | 109 | 42 | 651 | Complete | Oh et al., 2007 |
| Nile tilapia | *Oreochromis niloticus* | 50.0 | 28.0 | 12.0 | 7 | 112 | 35 | 560 | Partial | Elbialy et al., 2022 |
| Atlantic cod | *Gadus morhua* | 1035 | 9.90 | 0.00 | 30 | 396 | 64 | 634 | Partial | Bélanger et al., 2002 |

IBW = initial body weight; WT = water temperature; ADD = accumulated degree days; CG = compensatory growth.

^1^ADD (°C·d) = [Water temperature (°C) – Feeding cessation temperature (°C)] × Fasting or refeeding duration (d).


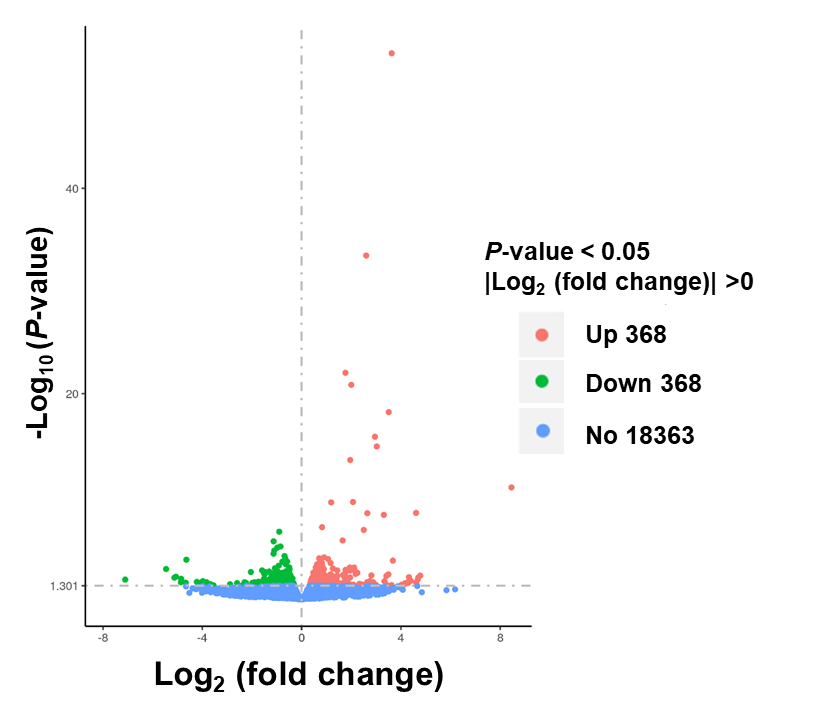


**Fig. S1** Volcano plot of differentially expressed genes (DEGs) between the FT12 and CON groups. The CON was the control group continuously fed, while the FT12 group was subjected to fasting for 12 d, respectively, followed by a 60-d refeeding (*n* = 3).
